# Supplementary material for: Genes Encoding Structurally Conserved Serpins in the Wheat Genome: Identification and Expression Profiles during Plant Development and Abiotic and Biotic Stress
Source: Int J Mol Sci. 2023 Jan 31;24(3):2707. doi: 10.3390/ijms24032707 (PMC9917288; doi:10.3390/ijms24032707)
Supplement: Supplementary file 1 [file ijms-24-02707-s001.zip › File S3_DNA seq alignment of WSZ1a.docx.docx]

**Supplementary File S3**

Alignment of sequences of the cDNA encoding WSZ1a, ESTs encoding WSZ1a, annotated wheat genes encoding WSZ1a, and the PCR product.

10 20 30 40 50 60 70 80 90 100

**cDNA-(Z49890)**  ATGGCAACCA CCCTCGCCAC CGACGTCCGC CTCTCCATCG CGCACCAGAC CCGCTTCGCC CTCCGTCTCG CCTCCACCAT CTCATCCAAC CCTAAGTCTG

**EST-CJ632731**  ATGGCAACCA CCCTCGCCAC CGACGTCCGC CTCTCCATCG CGCACCAGAC CCGCTTCGCC CTCCGTCTCG CCTCCACCAT CTCATCCAAC CCTAAGTCTG

**EST-CJ635636**  ~~~~~~~~~~ ~~~~~~~~~~ ~~~~~~~~~~ ~~~~~~~~~~ ~~~~~~~~~~ ~~~~~~~~~~ ~~~~~~~~~~ ~~~~~~~~~~ ~~~~~~~~~~ ~~~~~~~~~~

**TraesCS5B02G545000LC** ATGGCAACCA CCCTCGCCAC CGACGTCCGC CTCTCCATCG CGCACCAGAC CCGCTTCGCC CTCCGTCTCG CCTCCACCAT CTCATCCAAC CCTAAGTCTG

**PCR-seq-F**  ~~~~~~~~~~ ~~~~~~~~~~ ~~~~~~~~~~ ~~~~~~~~~~ ~~~~~~~~~~ ~~~~~~~~~~ ~~~~~~~~~~ ~~~~~~~~~~ ~~~~~~~~~~ ~~~~~~~~~~

**TraesCS5B02G362000.1** ~~~~~~~~~~ ~~~~~~~~~~ ~~~~~~~~~~ ~~~~~~~~~~ ~~~~~~~~~~ ~~~~~~~~~~ ~~~~~~~~~~ ~~~~~~~~~~ ~~~~~~~~~~ ~~~~~~~~~~

**PCR-seq-R**  ~~~~~~~~~~ ~~~~~~~~~~ ~~~~~~~~~~ ~~~~~~~~~~ ~~~~~~~~~~ ~~~~~~~~~~ ~~~~~~~~~~ ~~~~~~~~~~ ~~~~~~~~~~ ~~~~~~~~~~

110 120 130 140 150 160 170 180 190 200

**cDNA-(Z49890)**  CTGCCAGCAA TGCCGCCTTC TCCCCGGTCT CCCTCTACTC CGCACTTAGC CTCCTCGCCG CTGGCGCGGG CAGTGCCACT CGGGACCAAC TCGTCGCCAC

**EST-CJ632731**  CTGCCAGCAA TGCCGCCTTC TCCCCGGTCT CCCTCTACTC CGCACTTAGC CTCCTCGCCG CTGGCGCGGG CAGTGCCACT CGGGACCAAC TCGTCGCCAC

**EST-CJ635636**  ~~~~~~~~~~ ~~~~~~~~~~ ~~~~~~~~~~ ~~~~~~~~~~ ~~~~~~~~~~ ~~~~~~~~~~ ~~~~~~~~~~ ~~~~~~~~~~ ~~~~~~~~~~ ~~~~~~~~~~

**TraesCS5B02G545000LC** CTGCCAGCAA TGCCGCCTTC TCCCCGGTCT CCCTCTACTC CGCACTTAGC CTCCTCGCCG CTGGCGCGGG CAGTGCCACT CGGGACCAAC TCGTCGCCAC

**PCR-seq-F**  ~~~~~~~~~~ ~~~~~~~~~~ ~~~~~~~~~~ ~~~~~~~~~~ ~~~~~~~~~~ ~~~~~~~~~~ ~~~~~~~~~~ ~~~~~~~~~T CGGGACCAAC TCGTCGCCAC

**TraesCS5B02G362000.1** ~~~~~~~~~~ ~~~~~~~~~~ ~~~~~~~~~~ ~~~~~~~~~~ ~~~~~~~~~~ ~~~~~~~~~~ ~~~~~~~~~~ ~~~~~~~~~~ ~~~~~~~~~~ ~~~~~~~~~~

**PCR-seq-R**  ~~~~~~~~~~ ~~~~~~~~~~ ~~~~~~~~~~ ~~~~~~~~~~ ~~~~~~~~~~ ~~~~~~~~~~ ~~~~~~~~~~ ~~~~~~~~~~ ~~~~~~~~~~ ~~~~~~~~~~

210 220 230 240 250 260 270 280 290 300

**cDNA-(Z49890)**  ACTCGGGACA GGCAAGGTCG AGGGACTCCA CGCGCTCGCC GAGCAGGTGG TCCAGTTTGT GCTCGCCGAC GCGTCCTCCA CTGGTGGTCC GCGTGTCGCC

**EST-CJ632731**  ACTCGGGACA GGCAAGGTCG AGGGACTCCA CGCGCTCGCC GAGCAGGTGG TCCAGTTTGT GCTCGCCGAC GCGTCCTCCA CTGGTGGTCC GCGTGTCGCC

**EST-CJ635636**  ~~~~~~~~~~ ~~~~~~~~~~ ~~~~~~~~~~ ~~~~~~~~~~ ~~~~~~~~~~ ~~~~~~~~~~ ~~~~~~~~~~ ~~~~~~~~~~ ~~~~~~~~~~ ~~~~~~~~~~

**TraesCS5B02G545000LC** ACTCGGGACA GGCAAGGTCG AGGGACTCCA CGCGCTCGCC GAGCAGGTGG TCCAGTTTGT GCTCGCCGAC GCGTCCTCCA CTGGTGGTCC GCNNNNNNNN

**PCR-seq-F**  ACTCGGGACA GGCGAGGTCG AGGGACTCCA CGCGCTCGCC GAGCAGGTGG TCCAGTTTGT GCTCGCCGAC GCGTCCTCca CTGGTGGTCC GCGTGTCGCC

**TraesCS5B02G362000.1** ~~~~~~~~~~ ~~~~~~~~~~ ~~~~~~~~~~ ~~~~~~~~~~ ~~~~~~~~~~ ~~~~~~~~~~ ~~~~~~~~~~ ~~~~~~~~~~ ~~~~~~~~~~ ~~~~~~~~~~

**PCR-seq-R**  ~~~~~~~~~~ ~~~~~~~~~~ ~~~~~~~~~~ ~~~~~~~~~~ ~~~~~~~~~~ ~~~~~~~~~~ ~~~~~~~~~~ ~~~~~~~~~~ ~~~~~~~~~~ ~~~~~~~~~~

310 320 330 340 350 360 370 380 390 400

**cDNA-(Z49890)**  TTCGCCAATG GCGTCTTCGT GGACGCGTCG CTGCTCCTCA AGGCCATCCTT CAAGAGATT GCAGTGTGCA AGTACAAGGC CGAGACCCAA TCCGTGGACT

**EST-CJ632731**  TTCGCCAATG GCGTCTTCGT GGACGCGTCG CTGCTCCTCA AGCCATCCTT CCAAGAGATT GCAGTGTGCA AGTACAAGGC CGAGACCCAA TCCGTGGACT

**EST-CJ635636**  ~~~~~~~~~~ ~~~~~~~~~~ ~~~~~~~~~~ ~~~~~~~~~~ ~~~~~~~~~~ ~~~~~~~~~~ ~~~~~~~~~~ ~~~~~~~~~~ ~~~~~~~~~~ ~~~~~~~~~~

**TraesCS5B02G545000LC** NNNNNNNNNN NNNNNNNNNN NNNNNNNNNN NNNNNNNNNN NNNNNNNNNN NNNNNNNNNN NNNNNNNNNN NNNNNNNNNN NNNNNNNNNN NNNNNNNNNN

**PCR-seq-F**  TTCGCCAATG GCGTCTTCGT GGACGCGTCG CTGCTCCTCA AGCCATCCTT CCAAGAGATT GCAGTGTGCA AGTACAAGGC CGAGACCCAA TCCgTGGACT

**TraesCS5B02G362000.1** ~~~~~~~~~~ ~~~~~~~~~~ ~~~~~~~~~~ ~~~~~~~~~~ ~~~~~~~~~~ ~~~~~~~~~~ ~~~~~~~~~~ ~~~~~~~~~~ ~~~~~~~~~~ ~~~~~~~~~~

**PCR-seq-R**  ~~~~~~~~~~ ~~~~~~~~~~ ~~~~~~~~~~ ~~~~~~~~~~ ~~~~~~~~~~ ~~~~~~~~~~ ~~~~~~~~~~ ~~~~~~~~~~ ~~~~~~~~~~ ~~~~~~~~~~

410 420 430 440 450 460 470 480 490 500

**cDNA-(Z49890)**  TCCAAACTAA G~~~~~~~~~ ~~~~~~~~~~ ~~~~~~~~~~ ~~~~~~~~~~ ~~~~~~~~~~ ~~~~~~~~~~ ~~~~~~~~~~ ~~~~~~~~~~ ~~~~~~~~~~

**EST-CJ632731**  TCCAAACTAA G~~~~~~~~~ ~~~~~~~~~~ ~~~~~~~~~~ ~~~~~~~~~~ ~~~~~~~~~~ ~~~~~~~~~~ ~~~~~~~~~~ ~~~~~~~~~~ ~~~~~~~~~~

**EST-CJ635636**  ~~~~~~~~~~ ~~~~~~~~~~ ~~~~~~~~~~ ~~~~~~~~~~ ~~~~~~~~~~ ~~~~~~~~~~ ~~~~~~~~~~ ~~~~~~~~~~ ~~~~~~~~~~ ~~~~~~~~~~

**TraesCS5B02G545000LC** NNNNNNNNNN NNNNNNNNNN NNNNNNNNNN NNNNNNNNNN NNNNNNNNNN NNNNNNNNNN NNNNNNNNNN NNNNNNNNNN NNNNNNNNNN NNNNNNNNNN

**PCR-seq-F**  TCCAAACTAA G**GT**GACCCCC CCCCC..... .......... .......... .......... .......... .......... .......... ..........

**TraesCS5B02G362000.1** ~~~~~~~~~~ ~~~~~~~~~~ ~~~~~~~~~~ ~~~~~~~~~~ ~~~~~~~~~~ ~~~~~~~~~~ ~~~~~~~~~~ ~~~~~~~~~~ ~~~~~~~~~~ ~~~~~~~~~~

**PCR-seq-R**  ~~~~~~~~~~ ~~~~~~~~~~ ~~~~~~~~~~ ~~~~~~~~~~ ~~~~~~~~~~ ~~~~~~~~~~ ~~~~~~~~~~ ~~~~~~~~~~ ~~~~~~~~~~ ~~~~~~~~~~

510 520 530 540 550 560 570 580 590 600

**cDNA-(Z49890)**  ~~~~~~~~~~ ~~~~~~~~~~ ~~~~~~~~~~ ~~~~~~~~~~ ~~~~~~~~~~ ~~~~~~~~~~ ~~~~~~~~~~ ~~~~~~~~~~ ~~~~~~~~~~ ~~~~~~~~~~

**EST-CJ632731**  ~~~~~~~~~~ ~~~~~~~~~~ ~~~~~~~~~~ ~~~~~~~~~~ ~~~~~~~~~~ ~~~~~~~~~~ ~~~~~~~~~~ ~~~~~~~~~~ ~~~~~~~~~~ ~~~~~~~~~~

**EST-CJ635636**  ~~~~~~~~~~ ~~~~~~~~~~ ~~~~~~~~~~ ~~~~~~~~~~ ~~~~~~~~~~ ~~~~~~~~~~ ~~~~~~~~~~ ~~~~~~~~~~ ~~~~~~~~~~ ~~~~~~~~~~

**TraesCS5B02G545000LC** NNNNNNNNNN NNNNNNNNNN NNNNNNNNNN NNNNNNNNNN NNNNNNNNNN NNNNNNNNNN NNNAATGTAA AAGAATTTTT AACGGACATA ATTATGTACA

**PCR-seq-F**  .......... .......... .......... .......... .......... .......... .......... .......... .......... ..........

**TraesCS5B02G362000.1** ~~~~~~~~~~ ~~~~~~~~~~ ~~~~~~~~~~ ~~~~~~~~~~ ~~~~~~~~~~ ~~~~~~~~~~ ~~~~~~~~~~ ~~~~~~~~~~ ~~~~~~~~~~ ~~~~~~~~~~

**PCR-seq-R**  ~~~~~~~~~~ ~~~~~~~~~~ ~~~~~~~~~~ ~~~~~~~~~~ ~~~~~~~~~~ ~~~~~~~~~~ ~~~~~~~~~~ ~~~~~~~~~~ ~~~~~~~~~~ ~~~~~~~~~~

610 620 630 640 650 660 670 680 690 700

**cDNA-(Z49890)**  ~~~~~~~~~~ ~~~~~~~~~~ ~~~~~~~~~~ ~~~~~~~~~~ ~~~~~~~~~~ ~~~~~~~~~~ ~~~~~~~~~~ ~~~~~~~~~~ ~~~~~~~~~~ ~~~~~~~~~~

**EST-CJ632731**  ~~~~~~~~~~ ~~~~~~~~~~ ~~~~~~~~~~ ~~~~~~~~~~ ~~~~~~~~~~ ~~~~~~~~~~ ~~~~~~~~~~ ~~~~~~~~~~ ~~~~~~~~~~ ~~~~~~~~~~

**EST-CJ635636**  ~~~~~~~~~~ ~~~~~~~~~~ ~~~~~~~~~~ ~~~~~~~~~~ ~~~~~~~~~~ ~~~~~~~~~~ ~~~~~~~~~~ ~~~~~~~~~~ ~~~~~~~~~~ ~~~~~~~~~~

**TraesCS5B02G545000LC** TTTGA..... .......... .......... .......... .......... .......... .......... .......... .......... ..........

**PCR-seq-F**  .......... .......... .......... .......... .......... .......... .......... .......... .......... ..........

**TraesCS5B02G362000.1** ~~~~~~~~~~ ~~~~~~~~~~ ~~~~~~~~~~ ~~~~~~~~~~ ~~~~~~~~~~ ~~~~~~~~~~ ~~~~~~~~~~ ~~~~~~~~~~ ~~~~~~~~~~ ~~~~~~~~~~

**PCR-seq-R**  ~~~~~~~~~~ ~~~~~~~~~~ ~ATGTGTTTG AAAATTTTAC TATAAAAATG CTTTATAAAA ACACTAGTAT GCTGCTTTGC ATTCTTAGCA CAACTATTAT

710 720 730 740 750 760 770 780 790 800

**cDNA-(Z49890)**  ~~~~~~~~~~ ~~~~~~~~~~ ~~~~~~~~~~ ~~~~~~~~~~ ~~~~~~~~~~ ~~~~~~~~~~ ~~~~~~~~~~ ~~~~~~~~~~ ~~~~~~~~~~ ~~~~~~~~~~

**EST-CJ632731**  ~~~~~~~~~~ ~~~~~~~~~~ ~~~~~~~~~~ ~~~~~~~~~~ ~~~~~~~~~~ ~~~~~~~~~~ ~~~~~~~~~~ ~~~~~~~~~~ ~~~~~~~~~~ ~~~~~~~~~~

**EST-CJ635636**  ~~~~~~~~~~ ~~~~~~~~~~ ~~~~~~~~~~ ~~~~~~~~~~ ~~~~~~~~~~ ~~~~~~~~~~ ~~~~~~~~~~ ~~~~~~~~~~ ~~~~~~~~~~ ~~~~~~~~~~

**TraesCS5B02G545000LC** .......... .......... .......... .......... .......... .......... .......... .......... .......... ..........

**PCR-seq-F**  .......... .......... .......... .......... .......... .......... .......... .......... .......... ..........

**TraesCS5B02G362000.1** ~~~~~~~~~~ ~~~~~~~~~~ ~~~~~~~~~~ ~~~~~~~~~~ ~~~~~~~~~~ ~~~~~~~~~~ ~~~~~~~~~~ ~~~~~~~~~~ ~~~~~~~~~~ ~~~~~~~~~~

**PCR-seq-R**  ATTTTGTTCT TTGAAAGAAT TTTCTATTGG TTTGTTCAAA GGTTGGTGGC ACAGCAGTAC CGTCAAACTA TTTTCATTAC ATAATTGACC TTTGTTTATT

810 820 830 840 850 860 870 880 890 900

**cDNA-(Z49890)**  ~~~~~~~~~~ ~~~~~GCCGC TGAAGTTACT ACTCAAGTGA ATTCATGGGT AGAGAAAGTC ACGAGCGGTC GCATCAAGGA CATTCTCCCC CCAGGATCTA

**EST-CJ632731**  ~~~~~~~~~~ ~~~~~GCCGC TGAAGTTACT ACTCAAGTGA ATTCATGGGT AGAGAAAGTC ACGAGCGGTC GCATCAAGGA CATTCTCCCC CCAGGATCTA

**EST-CJ635636**  ~~~~~~~~~~ ~~~~~~~~~~ ~~~~~~~~~~ ~~~~~~~~~~ ~~~~~~~~~~ ~~~~~~~~~~ ~~~~~~~~~~ ~~~~~~~~~~ ~~~~~~~~~~ ~~~~~~~~~~

**TraesCS5B02G545000LC** .......... .......... .......... .......... .......... .......... .......... .......... .......... ..........

**PCR-seq-F**  .......... .......... .......... .......... .......... .......... .......... .......... .......... ..........

**TraesCS5B02G362000.1** ~~~~~~ATGG CCC**AG**GCCGC TGAAGTTACT ACTCAAGTGA ATTCATGGGT AGAGAAAGTC ACGAGCGGTC GCATCAAGGA CATTCTCCCC CCAGGATCTA

**PCR-seq-R**  TTTTCCATGG CCC**AG**GCCGC TGAAGTTACT ACTCAAGTGA ATTCATGGGT AGAGAA.... .......... .......... .......... ..........

910 920 930 940 950 960 970 980 990 1000

**cDNA-(Z49890)**  TTGACAATAC CACTAAACTT GTTCTTGCCA ATGCCCTTTA TTTCAAAGGA GCTTGGACAG AGCAGTTTGA TTCATATGGA ACAAAAAACG ACTACTTCTA

**EST-CJ632731**  TTGACAATAC CACTAAACTT GTTCTTGCCA ATGCCCTTTA TTTCAAAGGA GCTTGGACAG AGCAGTTTGA TTCATATGGA ACAAAAAACG ACTACTTCTA

**EST-CJ635636**  ~~~~~~~~~~ ~~~~~AACTT GTTCTTGCCA ATGCCCTTTA TTTCAAAGGA GCTTGGACAG AGCAGTTTGA TTCATATGGA ACAAAAAACG ACTACTTCTA

**TraesCS5B02G545000LC** .......... .......... .......... .......... .......... .......... .......... .......... .......... ..........

**PCR-seq-F**  .......... .......... .......... .......... .......... .......... .......... .......... .......... ..........

**TraesCS5B02G362000.1** TTGACAATAC CACTAAACTT GTTCTTGCCA ATGCCCTTTA TTTCAAAGGA GCTTGGACAG AGCAGTTTGA TTCATATGGA ACAAAAAACG ACTACTTCTA

**PCR-seq-R**  .......... .......... .......... .......... .......... .......... .......... .......... .......... ..........

1010 1020 1030 1040 1050 1060 1070 1080 1090 1100

**cDNA-(Z49890)**  CCTTCTTGAT GGGAGCTCAG TTCAAACACC ATTCATGTCC AGCATGGATG ACCAATACCT TTTGTCTTCT GATGGGTTGA AGGTACTTAA GCTACCTTAC

**EST-CJ632731**  CCTTCTTGAT GGGAGCTCAG TTCAAACACC ATTCATGTCC AGCATGGATG ACCAATACCT TTTGTCTTCT GATGGGTTGA AGGTACTTAA ----------

**EST-CJ635636**  CCTTCTTGAT GGGAGCTCAG TTCAAACACC ATTCATGTCC AGCATGGATG ACCAATACCT TTTGTCTTCT GATGGGTTGA AGGTACTTAA GCTACCTTAC

**TraesCS5B02G545000LC** .......... .......... .......... .......... .......... .......... .......... .......... .......... ..........

**PCR-seq-F**  .......... .......... .......... .......... .......... .......... .......... .......... .......... ..........

**TraesCS5B02G362000.1** CCTTCTTGAT GGGAGCTCAG TTCAAACACC ATTCATGTCC AGCATGGATG ACCAATACCT TTTGTCTTCT GATGGGTTGA AGGTACTTAA GCTACCTTAC

**PCR-seq-R**  .......... .......... .......... .......... .......... .......... .......... .......... .......... ..........

1110 1120 1130 1140 1150 1160 1170 1180 1190 1200

**cDNA-(Z49890)**  AAGCAAGGTG GGGACAATAG GCAGTTCTTC ATGTATATCC TTCTTCCAGA AGCACCAGGT GGTCTCTCAA GCTTGGCCGA AAAATTGAGT GCTGAACCAG

**EST-CJ632731**  ---------- ---------- ---------- ---------- ---------- ---------- ---------- ---------- ---------- ----------

**EST-CJ635636**  AAGCAAGGTG GGGACAATAG GCAGTTCTTC ATGTATATCC TTCTTCCAGA AGCACCAGGT GGTCTCTCAA GCTTGGCCGA AAAATTGAGT GCTGAACCAG

**TraesCS5B02G545000LC** .......... .......... .......... .......... .......... .......... .......... .......... .......... ..........

**PCR-seq-F**  .......... .......... .......... .......... .......... .......... .......... .......... .......... ..........

**TraesCS5B02G362000.1** AAGCAAGGTG GGGACAATAG GCAGTTCTTC ATGTATATCC TTCTTCCAGA AGCACCAGGT GGTCTCTCAA GCTTGGCCGA AAAATTGAGT GCTGAACCAG

**PCR-seq-R**  .......... .......... .......... .......... .......... .......... .......... .......... .......... ..........

1210 1220 1230 1240 1250 1260 1270 1280 1290 1300

**cDNA-(Z49890)**  ACTTCCTGGA GCGGCATATC CCAAGGCAGA GGGTTGCACT TAGGCAATTC AAGCTCCCCA AGTTCAAGAT ATCATTTGGA ATTGAAGCAT CTGATTTGCT

**EST-CJ632731**  ---------- ---------- ---------- ---------- ---------- ---------- ---------- ---------- ---------- ----------

**EST-CJ635636**  ACTTCCTGGA GCGGCATATC CCAAGGCAGA GGGTTGCACT TAGGCAATTC AAGCTCCCCA AGTTCAAGAT ATCATTTGGA ATTGAAGCAT CTGATTTGCT

**TraesCS5B02G545000LC** .......... .......... .......... .......... .......... .......... .......... .......... .......... ..........

**PCR-seq-F**  .......... .......... .......... .......... .......... .......... .......... .......... .......... ..........

**TraesCS5B02G362000.1** ACTTCCTGGA GCGGCATATC CCAAGGCAGA GGGTTGCACT TAGGCAATTC AAGCTCCCCA AGTTCAAGAT ATCATTTGGA ATTGAAGCAT CTGATTTGCT

**PCR-seq-R**  .......... .......... .......... .......... .......... .......... .......... .......... .......... ..........

1310 1320 1330 1340 1350 1360 1370 1380 1390 1400

**cDNA-(Z49890)**  CAAATGTTTG GGCCTCCAGC TTCCCTTCGG CGATGAAGCG GATTTTTCGG AGATGGTGGA TTCCCTAATG CCACAAGGTC TTCGTGTCTC ATCTGTTTTC

**EST-CJ632731**  ---------- ---------- ---------- ---------- ---------- ---------- ---------- ---------- ---------- ----------

**EST-CJ635636**  CAAATGTTTG GGCCTCCAGC TTCCCTTCGG CGATGAAGCG GATTTTTCGG AGATGGTGGA TTCCCTAATG CCACAAGGTC TTCGTGTCTC ATCTGTTTTC

**TraesCS5B02G545000LC** .......... .......... .......... .......... .......... .......... .......... .......... .......... ..........

**PCR-seq-F**  .......... .......... .......... .......... .......... .......... .......... .......... .......... ..........

**TraesCS5B02G362000.1** CAAATGTTTG GGCCTCCAGC TTCCCTTCGG CGATGAAGCG GATTTTTCGG AGATGGTGGA TTCCCTAATG CCACAAGGTC TTCGTGTCTC ATCTGTTTTC

**PCR-seq-R**  .......... .......... .......... .......... .......... .......... .......... .......... .......... ..........

1410 1420 1430 1440 1450 1460 1470 1480 1490 1500

**cDNA-(Z49890)**  CACCAAGCGT TTGTGGAAGT GAACGAACAA GGAACTGAGG CTGCAGCATC AACTGCTATT AAAATGGTCC TGCAACAGGC AAGGCCGCCC TCGGTCATGG

**EST-CJ632731**  ---------- ---------- ---------- ---------- ---------- ---------- ---------- ---------- ---------- ----------

**EST-CJ635636**  CACCAAGCGT TTGTGGAAGT GAACGAACAA GGAACTGAGG CTGCAGCATC AACTGCTATT AAAATGGTCC TGCAACAGGC AAGGCCGCCC TCGGTCATGG

**TraesCS5B02G545000LC** .......... .......... .......... .......... .......... .......... .......... .......... .......... ..........

**PCR-seq-F**  .......... .......... .......... .......... .......... .......... .......... .......... .......... ..........

**TraesCS5B02G362000.1** CACCAAGCGT TTGTGGAAGT GAACGAACAA GGAACTGAGG CTGCAGCATC AACTGCTATT AAAATGGTCC TGCAACAGGC AAGGCCGCCC TCGGTCATGG

**PCR-seq-R**  .......... .......... .......... .......... .......... .......... .......... .......... .......... ..........

1510 1520 1530 1540 1550 1560 1570 1580 1590 1600

**cDNA-(Z49890)**  ATTTCATCGC GGATCACCCT TTCCTCTTCC TTGTCCGGGA AGACATCTCT GGTGTGGTCC TATTCATGGG TCATGTGGTC AATCCCCTCT TATCTTCGTA

**EST-CJ632731**  ---------- ---------- ---------- ---------- ---------- ---------- ---------- ---------- ---------- ----------

**EST-CJ635636**  ATTTCATCGC GGATCACCCT TTCCTCTTCC TTGTCCGGGA AGACATCTCT GGTGTGGTCC TATTCATGGG TCATGTGGTC AATCCCCTCT TATCTTCNTA

**TraesCS5B02G545000LC** .......... .......... .......... .......... .......... .......... .......... .......... .......... ..........

**PCR-seq-F**  .......... .......... .......... .......... .......... .......... .......... .......... .......... ..........

**TraesCS5B02G362000.1** ATTTCATCGC GGATCACCCT TTCCTCTTCC TTGTCCGGGA AGACATCTCT GGTGTGGTCC TATTCATGGG TCATGTGGTC AATCCCCTCT TATCTTCGTA

**PCR-seq-R**  .......... .......... .......... .......... .......... .......... .......... .......... .......... ..........

**cDNA-(Z49890)**  A

**EST-CJ632731**  -

**EST-CJ635636**  A

**TraesCS5B02G545000LC** .

**PCR-seq-F**  .

**TraesCS5B02G362000.1** A

**PCR-seq-R**  .

Note 1: Errors in the original sequence of Z49890, a “G” insertion at 341 and a “C” deletion at 352, are shown in the alignment.

Note 2: Conserved splice sites “GT…” and “…AG” are highlighted in grey and bold-type in the PCR-fragment sequences (PCR-seq-F and PCR-seq-R). PCR primers were WSZ1a-F: CCCGGTCTCCCTCTACTCC and WSZ1a-R: GTGGTATTGTCAATAGATCCTGGG. Wheat Chinese Spring genomic DNA was used as template. The PCR product was sequenced from both primers.
