# Supplementary material for: Genes Encoding Structurally Conserved Serpins in the Wheat Genome: Identification and Expression Profiles during Plant Development and Abiotic and Biotic Stress
Source: Int J Mol Sci. 2023 Jan 31;24(3):2707. doi: 10.3390/ijms24032707 (PMC9917288; doi:10.3390/ijms24032707)
Supplement: Supplementary file 1 [file ijms-24-02707-s001.zip › File S1_All 83 putative serpin sequences listed in this study.docx.docx]

**Supplementary File S1**

>TraesCS1A02G289500

MSPLWKAVLCLTLFAAWRSQVEFRTMTSRRPRLDCAQRCPCSGQSYLHRPYSSSHVETQARAPLLTEAPPGGHSVSAAVGGHAGGKASCLPLAREAGVRAAAGGGGNFIVSPLSIHAALALVAAGTRGETREELLGFLGSASLDELHGAAATELAGRLNGLTQTSFACGVWVDRGQALEPEFMATAASGYAATAESVDFSSDPEQARRRVNAFVAGATDGRIRDVLPPGSVGSSTRVVLANALYFNGTWSQPFDQSATFTAPFHVPDGTIVRASFMTTGRFPFEQHVAVYPGFRALKLPYKNDGDQAGRRAEAAFYMLLLLPDGGAALGLADLYDKAVATPGFLKSHTPAVQVPVGRFMVPKFKFTFEFEASSDMQKLGVTRAFEGGDFSGMVSGGDGLFIAGVYHKATIEVDELGTVAAAATAVVIFQSARAPRPPVDFVANRPFLFAIVEEKTSAVLFLGHVINPLAE

>TraesCS1B02G299000

MSPLWKAVLCLTLFAAWRSQVEFRTMTSRRPRLDCAQRCPCSGQSYLHSSYSHAGTQARAPLLDEVLPGGHSVSVGAAIGGDEGGKASCLPLAREVGVRAAAGGGGNFIVSPLSIHAALALVAAGTRGETREELLGFLGSASLDELHGASSTDLAGRLNGLAQTSFACGVWVDRAQALEPEFMATAASRYAATAESVDFSSDPEQARRRVNGFVAGATDGRIRDVLPPGSVGSSTRVVLANALYFNGTWSQPFDQSATFTAPFHVPDGTIVRVPFMTTGRFPFEQHVAVYPGFRALKLPYKNDGDHAGRRAEAALYMLLLLPDGAALGLADLYDKAVATPGFIKSHTPGVQVPVGRFMVPKFKFTFEFEASSDMRRLGVTRAFEGGDFWGMVSGGDGLFIAGVYHKATIEVDELGTVAAAATAVAIQDSARAPRPPVDFVADRPFLFAIVEEKTSAVLFLGHVINPLAE

>TraesCS1D02G396900

MPRRNHATPCLLQVATMCPWGPRLRGPEGVVPATHQGGRRRGGRQGNQLHRLAAVHPRGARAGGRRHAGRDVWRAPGVPRVGVARRVARRAGHRAGAQAQRPGADVLRLRRVGRPEAGAGAGVRGHCRVKLRRHGATAEPVDFVQGAEQARRRVNAFVEGATSMRIRDILPPGSVGSSTVVVLANALYFKGAWFQPFDQSVTTVRVPFMTTGRFEFKQHIAVYPGFRALKLPYHNDGDHAAAAFYMLLLLPDDGSDLSLAGLYDKAAAAPEFIRSHAPAEQVPVGRFMVPKFKFTFEFEASADMRKLGVTRAFQGGDFSGMVSGGNGLSISGVYHKATIEVDELGTVAAAATAVSIPLSLSAPRPPVDFVADRPFLFAIAEEKTGGVLFLGHVVNPLAG

>TraesCS2A02G036700

MESMKEFALRRFPKLGRWFCADADAGADQAVPSDGLQAFSLGLNKRLAHDAGRRSNLVFSPLSVYAALSLVAAGARDRTLDELLGVLGAPSRDFLAGHVRALAEQALADQSKTGGPRVSFACGVWHDRTMPIRPAYRDAAESFKAVACAVNFRQKPEEAREEINAWVSASTDGLIPSILSRGALSDLTDLVLANAIYFKGKWAKPFAGYLTQHDKFHRLDGTAVDAAFMRGLGSHSIACHDGFKVLQLRYEEGHGPLLPQPPALAPTPAPIYSMCVFLPDARRGLWRLTDKIACNPDFLRKHLPRNSVLVGDFRLPKFKVTFDMTMNDVLQKMGVKEAFELGKADLSDMVEDGGRRKMALEKVIHRAVIEVNEEGTEAAAATCMTRLGCTPDSRPPAPCVDFVADHPFAFFIVEEVSGAILFAGHVLDPTIK

>TraesCS2A02G369100

MDKCMQVAWFAGTDAIARRSNFIFSPLSVRTGLALLATGTNGETLSQLLAFLGSQELHLLNAASASLVAEMRAWPQLTFAAGIFADKSFSLRPEFVSTASSAHRASVRSVDFQKQPAAAAAEVNALIAETTRGRIRDLVSPDSFQGDPKIVLANAMHFKATWARRFDPSDTVRRDFRRFDGTSVLVPFLSDPGMQYATSFDDLGFKVLQCFYKMAGRDGKLDPKAPLFSMLVFLPHRRDGLRDLLRLAVTEPDFVMRCAPRREQVVSPCLLPKFKFSFRFDATDALRGLGLAAPFDPLAADLSGAVSNMRPEGLYVSAIEQMCAVEVDEEGTTAVAAFYTHTSPTYSLCERPPPPPMSFVADHPFLFAIVEYGIGVVPRPRRGPFQLNSSVDGRFRFTREQSRAAMDEGPLRQHLFY

>TraesCS2B02G050210*

MESVKEFALRRFPKLGQWFYTEADADQAAPGDGLQAFSLGLNKRLAHDAGRRSNLVFSPLSVYAGLSLVAAGARDRTLDELLAVLGAPSRDFLAGHVRALAEQALTDQSKTGGPRVSFACGVWHDRTMPIRPAYRDAAESFKAVARAVNFRQKPEEAREEINAWVSASTDGLIPSILSRGALSDLTDLVLANAIYFKGKWAKPFAGYLTQDDKFHRLDGTAVDAPFMCGQGRHNIACHHGFKVLQLRYEEGHGPLLPQQPALAPTAAPIYSMCVFLPDARRGLWRLTDKIACNPDFLRKHLPRDTVLVGDFRLPKFKVTFDMTMNDVLQEMGVKEAFELGKADLSDMAEDGARRKLALEQVIHRAVIEVNEEGTEAAAATCMTRLGCTPHSLPLAPCVDFVADHPFAFFIVEEVSGAILFAGHVLDPTIK

>TraesCS2B02G325800

MAEQQVVDAMKDQAALSMRLLRGLGLRGEQNLAFSPASFHATLSLLAAGTAGAIRDQIVSFLGPAGAEAHAALVSHFGQTPSHQEEDEEGYPMVRCATGVWVDSSLRLKPTFATMAASRFNAEARAVCFGSSPEQARSEINEWFEGETGGRWKELVPEGSINAATVIVLANALYFKGWYDPFDPELTQDGDFYVSPGHAVRTPFMVGGYLHENMCIACHPGFKVLRMPYCGHYQDCRSSMCIYLPDDRGGLPELVRALSSDPSVLFAVPEKLVSTGELRIPKFDVSVRLEATQILRDLGLDLPFRLTPAGESFSEMLALDEHDSKMPMAVSSVVHQCSVNINEHGTVAAAATEMEILGFCLPEEKVVDFVADHPFLFFIIKEEDNNGVILFAGQVVNPLS

>TraesCS2B02G386300

MDQCLQVAWFAGTDAIARRSNFIISPLSVRTGLALLATGTSGETLTQLLAFLGSQDLHLLNAASASLIAEMRAWPQLTFAAGIFADKSFSLRPEFVSAATSAHRASVRSVDFQKQPAAAAAEVNALIAETTRGRIRDLVSPDSFRGDPKIVLANAMHFKATWARRFDPSDTVRCDFHRLDGTSVRVPFLSDPGMQYATSFDDLGFKVLQCFYKMAGRDGKLDPKAPLFSMLIFLPHRRDGLRDLLRLVVTEPDFVMRCAPRREQVVSPCLLPKFKFSFRFDATDALRGLGLAAPFDPLAADLSGAVSNMPREGLYVSAVEQMCAVEVDEEGTTAVAAFYTHTSPTYSPFERPPPPPPPMSFVADHPFLFAIVEYGKGEVLFLGHVVDPSS

>TraesCS2B02G386500

MDQCLQVALFAGTDAIARRSNFIFSPLSMRTGLALLATGTNGKTLSQLLAFLGSQDLHLLNAASASLIAEMRAWPQLTFAAGIFADKSFSLRPEFVSAAASAHRASVRSVDFQNQPAAAAAEVNALIAETTRGRIRDLVSPDSFRGDPKIVLANAMHFKATWARKFDPSDTVRRDFHRLDGTSVRVPFLSAPGMQYATSFDDLGFKVLQCFYKMAGRDGKLDPKAPLFSMLIFLPHRRDGLRDLLRLAVTEPDFVMRCAPRLQQVVNPCLLPKFKFSFRFDAMDALRSLGLAAPFDPLAADLSGAVSNMPREGLYVSTVEQMCAVEVDEEGTTAVAAFYAPSNPTYSPCERPPPPPMSFVADHPFLFAIVEYGKGEVLFLGHVVDPSS

>TraesCS2B02G530600

MEDLAGAIRDLAALSTRLLLQLSGDGEKRNLAISPLSIHSVVVLLAAGATGDTLDQIVSFLGLSGGAAHAALASEVATLVFGRDAGVEPQIQCAVGVWVESSLRLRSAFADKVTSEFKAGVRAMPFRENVEEARVEINRWFEDKTGGFIKDLMPEGHLDAISTVLVIGNALYMRGTWLDPFIPYNTLDDDFFLPDADGSRVRVPFMTSTNDQRISCHPGFKVLLLPYESKGNHEFSMHIYLPDERNGLQALVREISSSGTAEFLDRCVPTRHVAVRNLRIPKFQVSSKIDARDVLKGLGLELPFHFTYDWSEMIEFSEPAPPVAVQNVLHECVVEVNEDGTMAAAATEADLMMGFSITGEEEPAAHVDFVADHPFLFLIREDKSGIVLFAGQVVNPLL

>TraesCS2B02G530700

MEGLAGVISDLAALSTRLLLQLSGDGEKRNLAISPLSIQSVLVLLAAGATGDTLDQIVSFLGLSGGAAHAALASEVATLVFGRADGVEPEIRCAVGVWVESSLRLRSAFADKVASKFKAAVRAMPFRENVEEARVEINRWFEDKTGGFIKDPMPEGHLDATLTALVIGNALYMRGTWLDPFDPEYTQDGDFFLADTDGSRVRVPFMTSKNDQCISCHPGFKVLQLHYESKGGSNHRFSMHIYLPDERDGLEALLLEISSSGTAEFVARCVPAARRVEVGNLRIPKFKVSSKIDARDVLQGLGPELPFRFTHDRSEMIELAEPEPPLRVQNVLHECVVEVNEDGTMAAAATEADDDVGFSPYGEEPARVDFVADHPFLFLIREDKSGILLFAGQVLNPLL

>TraesCS2D02G036000*

MESMKEFALRRFPKLGRWFCTEADAGADQAVPSDGLQAFSLGLNKRLAHDAGRRSNLVFSPLSVYAGLSLVAAGARDRTLDELLVVLGAPSRDFLAGDVRALAEQALTDQSKTGGPRISFACGVWHDRTMPIRPAYRDAAESFKAVARAVNFRQKPEEATEEINAWVSASTDGLIPSILSRGALSDLTDLVLANAIYFKGKWAKPFAGYLTQHDKFHRLDGTAVDAPFMRGLGSHNIACHDGFKVLQLRYEEGHGPLLPQPPALAPTPVPIYSMCVFLPDARRGLWRLTDKIACNPDFLRKHLPRNSVLVGDFRLPKFKVTFGMTMNDVLQEMGVKEAFELGKADLSNMVENGGRRKMALEKVIHMAVIEVNEEGTEAAAATYMTRLGCTPDSRPPAPCVDFVADHPFAFFIVEEVSGAILFAGHVLDPTIK

>TraesCS2D02G102600

MDFRGGGPTRWHQPQPAVSAGSEGLAALSASLARALADKHANSNLVFSPLSIYTALALVAAGARGATLDEILLVLGVPSRGELDAFVARAAGAALQDQSGSGGPRVAFACGVWSDMACPLKPGFRRAVVDGPYGAEASTVDFRGDPEGSRQLINAWAARATNSLIDSVLGRNSVNELTRVVLGNAVYFKDKWDQPFHKSDTSDAPFRRAGGAGAVDVPFMQSWKRQFVAVHDGFKVLKLEYKMDTNDGLRSLLDAIASRSGFLHEHLPTQKVDVGEFRVPRFKLSFHDSVVGVLNKLGLRLPFSEAADLSDMTGLPLVLSEVIHKAVIEVNEEGTRAAAVTMEEGCAAKPRPPPPVDFVADHPFAYFIVDEGTGAVVFAGHVLDPSTQ

>TraesCS2D02G102700

MACPLKPGFRRAVVDGAYGAEASTVDFRGDPEGSRQLINAWAARATNDLIDSVLGKGSVSPLTRVVLGNAVYFKGKWEKPFEKRHIADAPFRRAGGPIDVPFMQSWKMQFVAVHAGFKVLKLKYEMGDARARPPLHPRDDASTQDRRTEIFHRDPTKPAPFFPNRAPLGHPDRFSHAARQPNASSRLAGLPSSSSSPTQFSMCIFLPDADDGLRSLLDAIASRPGFLHEHLPTQKVDVGEFRVPKFKLSFHDSIVGVLNKLGLGLPFSEAPDLSDMTEDDRSGLPLVLSEVVHKAVIEVNEEGTEAAALTMCQMFIGCAARLRLPPPVDFVADHPFAYFIVEEGTGAVVFAGHVLDPSTQ

>TraesCS2D02G170500

MYFEGKWEDQPFDRGHTAHKPFRRLDRSQVSVPFMQSWKPQFVAVHDGFKVLKLRYKMAAPDHEGTTREHAPPPPLGHGDHGVHGHGAPYAHRHVSAAYNDGGPSPNYLPTGPPPYFRHRRHDPPPWAGSPISYAHPRPYPYGALGNAYPSQGAAPWAHSGSDRTGYTQFSMCIFLPDAHDGLWGLLDTMASRPPGFLHDHLPEQRIALREFRMPKFKLSFHSSVAAVLKKLGLELPFCQQGNLSDMVEDDGSGLPIVVDDVIHKAVVEVNEEGTEAAAATMVVQGLGCAMPSPPTPPQVDFIADHPFAYYIVEEATGAVVFAGHVLGPSKE

>TraesCS2D02G307100

MEEQQVVDAMKDQAALSMRLLRGLGLRGEQNLAFSPASFHAILSLLAAGTTGAIRDQIVSFLGPAGAEAHAALVSHFGQTPSHQEEDEEGHPMVRCATGVWVDSSLHLKPTFATMAASRFNAEARAVCFGSNPEQARSEINEWFEGETGGRWKELVPEGSINAATVVVLANALYFRGYWYDPFDPELTQDGDFYVSPGHAVRTPFMVGGYLHENMCIACHPGFKVLWMPYCGHYQDCRSSMCIYLPDDRGGLPELVRALSSDPSVLFAVPEKLVPTAELRIPKFDVSQRLEASHLLRDLGLDLPFRLNPAGQSFSEMLALDEHDSKMPMAVSSVVHQCSVNINEQGTVAAAATDMEILGCCLPSEKVVDFVADHPFLFFIIKEEDNNGVILFAGQVVNPLS

>TraesCS2D02G365900

MDQCLQVALFAGTDAIARRSNFIFSPLSMRTGLALLATGTNGETLRQLLTFLGSQDLHLLNAASASLVAEMRAWPQLTFAAGIFADRSFSLRPEFVSGAASAHRASVRSVDFQNQPAAAAAEVNALIAETTRGRIRDLVSPDSFRGDPKIVLANAMHFKATWSRRFDRSDTTRSEFHRLDGTSVRAPFLSAPGMQYATSFDDLGFKVLQCFYKMAGRDGKLDPKAPLFSMLIFLRHRRDGLRDLLRLAVTEPDFVMRCAPRREQVVNPCLLPKFKFSFRFDATDALRGLGLAAPFDPLAADLSGAVSNMPPEGLYVSAVEQVCAVEVDEEGTTAVASFYAPTNPTYSPFERPPPPPMSFVADHPFLFAIIEYGKGEVLFLGHVVDPSS

>TraesCS2D02G373300

MEVTADTTPSCSGGLAALAASLTSRLAVTVKNRSSFLYAIFFPSQKQAMEATDAHYSKVTGDTAPSCSGGLAALAAGLVSRLADDNNAGSNLVLSPLSIYAALALLAAGARGATQDEILGVLGTPSRAALDEFLSGVAEDALEDHSEYGGPRVALRAAPEAARGQINAWVAQATSNLIGPVLGPGSITRLTRVVLGNAIYFKGKWEDPFYKEATANKLFHRPDGSTVDVPFMQSWSSQFIAVHKGFKVLKLRYQMAQGQGRHVDRIKRTQFSMCIFLPDAFDGLPSLVDAIASRPGFLHKHLPKRKLEVREFRVPKFKLSFHSSDVTVLKKLGLQLPFSDQADLSDMVERDESDLPLVLSDVIHKAVIEVNEEGTVAAAVSRMNMEIGSSITRRPPPPPVDFVADHPFAYFIVEEATDAVVFAGCVLDPSNED

>TraesCS3A02G300700

MELAEAAFAMRVLRHLACAGGTAAASGANLAVSPLSIHAALALLGAGARGATLDQVVAFLGPAHAALASHVALRLLSDSPGDDGGPSVRFANGVWVDAAMRLKVAYAALVSQHYRAQALPASFKDMPEEARTQINRWFESLTAGRIKGLLPQGSINGATLAVLGNALYFKGAWCRKFDPRLTLDDTFHLPAGGSVRAPFMSSSDRQQHVACRSGYKVLRLPYARGRERRYFSMYIYLPDERDGLQSLLHRLGSDPALLESSTTLTAQVPVGAFKVPRFTISCKTDATELLQDLGLRLPFAPLAADFSEMLDSAAPLVVSAVFHQSFVEVNEEGTEAAAASAVVASFGAAAVRTPVQVVDFVADHPFVFLIKEELSGVVVFAGQVINPLVP

>TraesCS3A02G504800

MGSDGLTAFALRLAKKLGEGDNTRGSNIAFSPLSLYTTLGLVAAGACGRTLDELLALLGAASADELAGFVHGLPSDPSGSGGPVITYTYGVFHQEHMELTPDFLHTATESYKAEIRAVDFAEDEVREETRKEINQWAAAETNNLIWEFLPEGSLTDHSRFVLTNAIYFKGAWETRFPENLTEDHEFHRLDGADPVDVPFMTLPGTCELFVSYNEGFKVLKLPYKAGDDAMSRYSMCVFLPDEDDGLHAMVSTLADMGGSLLDHVPKLRSRVRELMLPKFKLSFFCRLAQVLRGLGLREAFTEEAADLSGIMDKSVCDVRLDEVFHKAVVEVNEEGTVAAACAAVIGRKKKCAMRLEFIADHPFAFYIVEEVSGAVVFAGHVLDPSSSP

>TraesCS3B02G335800

MELAEAARDEAAFAMRVLRHLACGGGGGKAGANLAVSPLSIHAALALLGAGARGATLDQIVAFLGPAGGSAHAALASHVALRLLSDSPSDDGGPSVRFANGVWVDAAMGLKVDYAAVVSQHYRAQALPASFKDMPEEARTQINRWFESVTAGRIKDLLPQGSVNGATLAVLGNALYFKGAWCRKFDPRLTLDDAFYLPAGGSVRALFMSSRDRQQHIACRFGYKVLKLPYARGRERRHFSMYIYLPDERDGLQSLLHRLGSDPALLESSTTLMAQVPVGAFKVPKFTISCKTNATELLQDLGLRLPFAPLAADFSEMLDSAAPLVVSAVFHQSFVEVNEEGTEAAAATAVVASFGAAAVRTPVQVVDFVADHPFMFLIKEELSGVVVFAGQVIDPLVP

>TraesCS3B02G520000

MELAEAVRCEAAFGMRVLQHLAAEPGAGGKNHAVSPLSIHAALALLGAGARGAMLNEIVALLGPAGGRAHALLASHVAMHVFADSSGGDGGPKVQFANAVWVDATAAPLKADYARVVAQHYAFKTMPEEARREINEWFEAATAGRIKEFLPQGSVGYDTAAILGNALYFKGVWESTFDARLTRHDTFFYQQPAGGEGQIRVPFMSSGERQYIACRPDYKVLKLLYACGSGEHRRRFAMHVYLPNERHGLQAMLHRLASSPEQLEADSMALRTTVAVGTFKVPKFTISYKTEASGMLQRLGLRLTFSTASDFSGLLDLEHMKPPRLPLYVSQVYHESFVEVNEEGTEAAAATAIVGIFGSCSAVCSRPVNYVDFIADHPFMFLIKEELTGVVVFAGQVVDPSL

>TraesCS3B02G520200

MEPEVAEAARGEAAFSIRALHHIALAAGASASSKNIAVSPVSIHAAVMLLGTAARGATLDEIVAFLGPAGGRAHALLASDVALRVLAHSAGDHGGPKVRFANSVWVDAASARLKADYAGVVADQFRAQAYAASFTTKPEEARRDINRWVQAATAGRIKGFLPQGSVGAGTRVILANALYFKGVWESKFDARLTQQHRFYLLNGGHVRVPFMSSGESQHIACCPEWKVLKLHYAPGRRGVEHLRRHFAMYVYLPNELHGLPSMVRKLASSPELLEAGSMDLRDMVPVRDFRLPKFTVSYKTEATGLLQGLGLRLPFDDDAADFSEMLEDSKGSLVVSNVYHQSLVEVNEEGTEAAATTAVTSMTFTCPAVMSRPVKYVDFVADHPFMFLTKEELTGVVVFAGLVLDPSS

>TraesCS3B02G567700

MADQEPERPSKKARRSPPAMDSGGLTAFALRLAKKLAEGDDTRSSNIAFSPLSLYTTLSLVAAGARGRTLDQLLGLLGAAPPDELAGFVRGLTADLSGNGGPVITYAYGVFHQKHMELTPDFLRTATESYNAEIRAVDFAEGDRKKIRKEINKWAAAATNNLISEILPERSLSQLSRFVLTNAIYFKGVWETRFPENLTEKHEFHRLGDADPVDVPFMTPPGECKLFVSHGKGFKVLKLPYKVATPAAMSSRYSMCVFLPDDNDGLHAMVTALAEAGGSLLDHVPKHRSSVRKLMLPKFKMSFFCRLTKVLQGLGLRDAFSESADLSGLATKIDCDVRLDEVFHKAVLEVNEEGTVAAACTAVTGRVKQTARRPLQFIADHPFAFYIVEEVSGSVVFAGHVLDPSSSQ

>TraesCS3B02G567900

MGDQEAEWLSKKAGGSSPPPAMGSDSLTAFALRLAKKLAEGDDTRGSNIAFSPLSLYTTLGLVAAGARNRTLDELLALLGAASPDELAGFVRGLPADPSGSGGPVITYAYGVFHQKHMELTPDFLHTATESYNAEIRAVDFAEEEVREETRKEINQWAVAVTNNLISEILPEGSLTDLSRFVLTNAIYFKGVWETGFPKSHTEEHKFHRLDGADPVNVPFMTLPGTCELFVSYNEGFKVLKLPYKAGEDAMSRYSMCVFLPDEDNGLHAMLSTLEEVGGSLLDHVPKHRRRVRELMLPKFKMSFFCRLAQVLRGLGLREAFTEEAADLSGIVEKSVCDVRLDEVFHKAVIEVNEEGTVAAACAAVISRKKKSAMRLEFIADHPFAFYIVQEVSGAVVFAGHVLDPSSSQ

>TraesCS3D02G301100

MELTEAARDEAAFAMRVLRHLACGGGKASASGANLAVSPLSIHAALALLGAGARGATLDQVVAFLGPAGGPAHAALASHVALRLLSDSPGDDGGPSVRFANGVWVDAAMRLKVDYAALVSEHYRAQALPASFKDMPEEARTQINRWFESATAGRIKGLLPEGSVNGATLAVLGNALYFKGAWCRKFDPRLTLDDTFHLPAGGSVRAPFMSSRDRQQHVACRSGYKVLQLPYARGRERRYFSMYIYLPDERDGLQSLLHRLGSDPALLESSTTLTAQVPVGAFKVPKFTISCKTNATELLQDLGLRLPFAPLAADFSEMLDSAAPLVVSAVFHQSFVEVNEEGTEAAAATAVVASFGAAAVRTPVQVVDFVADHPFMFLIKEELSGVVVFSGQVVNPLVP

>TraesCS3D02G511400

MADQEEARPTKKARGSSPPPAMGSGSLTAFALRLAKKLAEGDDTMNSNIAFSPLSLYTTLGLVAAGARGRTLDELLALLGAASADEVARFVRGLAADPSGSGGPIITYAYGVFHQKHKELTPDFLHTATESYSAEIRAVDFAKDEVREETRKEINLWAAAATNNLILEILPEGSLTDLSRFVLTNAIYFKGAWETRFPKKLTEDREFYRLDGADPVEVPFMTLPGECQLFVSYNEGFKVLKLPYKAGDDAMSRYSMCVFLPDEEEGLHDMVRSLEEVGGSLLDHVPMYHSSVREILLPMFKLSFFCGLSKVLRGLGVQEAFSKEADLSGIMEKSVCDVRLDEVFHKAVVEVNEEGTVAAACTAVVGRKKQCARRSLEFIADHPFAFYIVEEVSGAVVFAGHVLDPSSSQ

>TraesCS4A02G205200

MRHFSSLPRVLRRSAAPKPNRRSPAPKPHPPSPSPGPVPDAQAPVAPAPMPTRPWEEALDVAQRAFCLPLAGRVLAAAGTGNAAVSPVGVHAALSLAASGARGATRRQLLGTLGCGGGGKGAAADAANVASRVVKRVLKDRAKSGGPRLAFASGVWADASTTLSPEFVETAGGLYCSAAKTVDFKSTPEDAAEQINSWVNKSTRQTITSLVPDGLVDQNTGLVLGSALYFKGRWLDKTDIGKTAEQKFYCLDGTHVLVPFVEYDRTRLFAAHDGFKVIKLPYKQGNNERKFSMYIFLPDAHDGLFELTKKIFSEPAFLEQHLPTEKRHVGIGVPKFTISFQIDMKDFLKDMTLELPFRRDADFKDMVKEGDSKEPLFLSDVLHKVILEVNDDEIEETSVEKSIGKPLPTEHFTADHPFFFLIREEVSATVIFMGHVLDPSSQY

>TraesCS4A02G235700

MATTDIRLSIGHQTRFALRLASAISSPSHAKGSAGNAAFSPLSLHVALSLVAAGAGGATRDQLAAALGSAEKGGSEDLHALAEQVVQVVLADASGAGGPRVAFANGVFVDASLKLKPSFKDLAVGKYKAETQSVDFQTKAAEVAGQVNSWVDKITTGLIKEILPAGSVDNTTRLVLGNALYFKGAWTEKFDASKTKDEKFHLLDGSSVQTPFMSSTKKQYISCSDSLKVLKLPYQQGEDKRQFSMYILLPEAQDGLWNLANKLSTEPEFLENHIPMQKVPVGQFKLPKFKILFGFEASDMLKGLGLQLPFSAEADLSEMVDSSAGLYVSSVFHKSFVEVNEEGTEAAAATASVVTLRSLPVEPVKVDFVADHPFLFLIREDLTGVVLFVGHVFNPLVSP

>TraesCS4A02G235900

MAAADIRLSVAHQTRFALRLASAISSPSNADGAAGNAAFSPLSLHVALSLVAAGAGGATRDQLAATLGAEGPGEAESLHALAQQVVQLVLADASVEGGPRVAFANGVFVDTSTPLRSSFKEVAVGKYKAETHSVDFQTKAAEVACQVNTWVEKLTSGLIKEILPDGSVDSTTRLVLGNALYFKGAWTEEFDASKTKDGEFHLLDGSSVQASFMSSTNEQYLSSHDNLKVLKLPYRQGGDMRQFSMYILLPEANDGLWNLAGKVSSEPEFLEKHTPTEKVLVRNFKLPKFKISFGFEASSLLKGLGLHLPFSCEADLSEMVDSAAEQNLYISSVFHKSFVEVNEEGTEAAAATAAVVMLMSLPLDPPMEMDFVADHPFLFVIREDLTGVVLFVGHVVNPLLAA

>TraesCS4A02G422200

MVGQAVADAVMDQAALCLLLLRHLGSLDEGMPTPTTNLAFSPISFHAVLSLLAAGASGATRDQIVTFLGPAGAEAHAALASKVASFVLAGRGLAQAHGATGVWVDASLRLSPTFADTAAAVYKADARSVDFSNKPREAMAEINEWFESKTRGLVKNIVSERDCDGSTALVLGNSVFFSGYWNSPFFPGATEEGRFYVNASPEDHTVRVPFMKGSPFQQIGVHPGFKVLRMPYRGDFGDSDPLFAMYIYLPDDRDGLPALARTLSASPAALLHRSVVPEQPVPLGELKIPKFEVSVRVEASRMLRNLGLELPFRRSRDSFSEMLSPPAPPVAVSSVVHQCVVKVDERGTVAAAGTVAMSAGFGMVRDRPVDFVADHPFAFFLMEDVSGVVVFAGHVVNPLSH

>TraesCS4A02G427400

MAADALQDRSASGGPRVAFACGVWSDLSRPLKPAFREAVVGTYKAEASTVNFQGAPEETRGQINAWAAQVTRNLIRNVLPKGSINPATTRVVLGNAMYFKGKWEDQPFDRRYTVHEPFHRLARSQVAVPFMQSTMSQFVAVHDGFKVLKLRYKMVAPDHEEHVHPPPYLGLILIVPIPWCNHGRVGQSGEYGGPDHGAPYSHAHVRASYNRRGRSANYISTGPPPYFRHRPYPPPPFRNPTSPYAYPRPYPNGALGRFGHPPNPPYIALPHVPGRTGYPGNAFGNRYKPDPWAGNAYPSQCAARSVFSGSNRTGYTQFSMCIFLPDAHDGLWGLLDTIASRPGFLHDHLPEQQIALREFRMPKFKLSFHSSIVAVLKKLGLELPFCERGNLSEMVEGDGSGLPIVVGDVIHKAVVEVNEEGTEAAAVTVLVGALGCARPPSPPTPPQVDFIADHPFAYYIVEEATGAVVFAGHVLDPSKE

>TraesCS4A02G436000

MVGQDVADAVKDQAALCLLLLRHLGSLDEGMPTTNLAFSPISFHAVLSLLAAGASGATRDQIVTFLGPAGAEAHAALASMVASFVLAGRGLAQSHGATGVWVDASLRLNPTFANTAAAVYQADARSVDFSNRPREATAEINEWFESKTRGLTKNILSEHDCDGSTSLVVGNSVFFSGHWNAPFFPEATEEGPFYVNASPEHTVRVPFMKGSPFQQVGVHPGFKVLRMPYRGDGGDGEPLFAMYIYLPDNLDGLPALARRISASPDALLHRSVVPEQVVPLGKLRIPKFEVTLRVEASQMLRNLGLELPFRRSGDSFSEMLSPPAAPVAVSSVVHHCVVKVDERGTVAAAGTVAMSAGFGMVRDRPVDFVADHPFAFFLMEDVGGVVIFAGHVINPLPLMDGLPALL

>TraesCS4B02G079100

MAATDTRLSVAHQTRFALRLASAISSPSNADGAAGNAAFSPLSLHVALSLIAAGAGGATRDQLAATLGAVGQGEAESLHALAQQVVQLVLADASVEGGPRVAFANGVFVDTSTPLKSSFKEVAVGKYKAETHSVDFQTKAGEVACQVNTWVEKITSGLIKEILADGSVDSTSRLVLGNALYFKGAWTEEFDASKTKDGKFHLLDGSSVQAPFMSSTNEQYISSCDNMKVLKLPYRQGGDMRQFSMYILLPEANDGLWNLAEKVSSEPEFLEKHTPTEKVPVRHFKLPKFKISFGFEASSLLKGLGLHLPFSGEADLSEMVDSAEEQNLCVSSVFHKSFVEVNEKGTEAAAATAAVVMLESLPLDPPMEIDFIADHPFLFMIREDLTGVVLFVGHVVNPLLAA

>TraesCS4B02G079200

MATTDIRLSIGHQTRFALRLASAISSPSHAKGSAGNAAFSPLSLHVALSLVAAGAGGATRDQLAATLGAAEKGGAEGIHALAEQVVQVVLADASGAGGPRVAFANGVFVDASLKLKPSFKDLAVGKYKAETQSVDFQTKAAEVAGQVNSWVEKITTGLIKEILPSGSVDNTTRLVLGNALYFKGAWTEKFDASKTKDEKFHLLDGSSVQTPFMSSTKKQYISCSDSLKVLKLPYQQGEDKRQFSMYILLPEAQDGLWNLANKLSTEPEFLENHIPVQKVPVGQFKLPKFKVSFGFEASDMLKGLGLQLPFSKEADLSEMVDSPAGLYVSSVFHKSFVEVNEEGTEAAAATAAVVTLRSLPVEPVKVDFVADHPFLFLIREDITGVVLFVGHVFNPLVSP

>TraesCS4B02G093000

MTGGLEARPSKKRRQSGSGLAEFVTRITKHLADASPRGNMVFSPLSIYAAVALLAPGARGETLDEILRLLGVHSRDELEDLISRVAADALKDQSQSGGPSVAFACGVWNDKARPLKTAYRETIVGTYKAKSRAVDFRGNAGKAARRINAWAARVTGNLITAIVSPKSFGPATDVVLGNAIYFKGKWDLPFYQRMTKDRPFHRLDGTTVDAPFMRNSGRHFIAVYDGFKVLKLRYEMPRAKPLGWTVNFKKTTPRYSMCIFLPDAYDGLQGLVEEITSRPTFLHEHLPTSQVRVGEFGVPKFKLSFQSSVIQTLKHLGLLLPFGMGADLSDMVEDDGSGLPLVVNDVFHKAVIEVNEQGTEAAADTTTKIYGLAMPRPTPTTDFVADHPFAYFIFEEASGAIIFAGHVVDPSSGGDSQVRIGRPRKG

>TraesCS4B02G093900

MRRDPPQSAGSRGLAALSASLARGIAGNPVDSNLVFSPLSIYTALALVAAGARGATLDEILRVLGARSRGELDEFVARAVGGALRDRTDSGGPRVAFACGVWSDLACPLKPGFLKAVVDGGYRAEASTVDFRGDPDGSRQLINAWAARATNNLIDSVLGPRSVTESTRVVLGNAVYFKGKWDQPFDERNTADAPFRRLGGALPVDVPFMQSWDEQFVVVHDGFKVLKLRYKMVDALLTRDPKKPAPFFPKRAPLLRPDRFSHAASPSRHGSYGRAGPYPYSNGHPNASSSLAGSYGGNNTSSTASSNNNSNNFNSSSTHFSLCIFLPDADDGLRSLVDAIASRPGFLRDHLPRRKVEVGEFRVPRFKLSFHDSVVDVLKELGLSLPFSPLGDLSDMTGPDDSGFGMIVDEVVHKAVIEVNEEGTEAAAVTMVTDRFGCAARCNPPPQVDFVADHPFAYFIVEEESGAIVFAGHVVDPSRES

>TraesCS4B02G229800

MDTAAMAGAMRDLAALSMRFLLHLSGNQTNLAFSPLSFYYVLVLLAAGATGDTLNQIVSFLGPSGGMAHASLATHAASAFLARGNGSEPDVRCGVGVWVDSSLQLRPAFADMVASRYNATAQAMPFQEKPDEARVEINRWFEDKTGGLIKELMPEGHLDSDTALVIGTALYLRGSWLRPFDREDTVDGDFFLANGSSVRVPFMTSGNRQRISSHAGFKVLQLPYDSGRVGGRQSFSMHIYLPDERDGLQALIRELSSDTAGFLNRSAPAQAVEVGDFKVPKFKVSRKVDVSDLLKDMGLERPFCFSHDFAEMVDYSEPLAVRSVLHECVVEVDEDGTMAAAATEADIMTGCSIGWEEPVRVDFVADHPFLFLIREDESGIVLFAGQVVNPEL

>TraesCS4B02G309900

MQSVKELVLRCLWLCTEAEAKPDHALAASDGLQAFAPAASDGLKAFALGLNKRLTDDDAGRSGNLVTSPLSVYAALALVAAGAREGTLDELLRVLGAPSLDFLAGHVRALGEHALADGSRTGGPRVSLACSVWHDVTMPLRPAYRATAAESYKAVARAVNFRQKPEEAREQINAWVAAATNDLIPSILSPDALSSLTVLVLANAIYFKGMWEKPFDKELTKDDKFHCLDGIAVDAPFVRGLGWHYIACHDGFKVLQLRYVQGHSSQGQPQPPPIYSMCVFLPDTRDGLWELTDKIASNPDFVRKHLPCGDVMVSDFRLPKFKVNFGMTMEGILQDMGLNEAFEPGKADLSDMAEDGTGKLALEKIIHRAIIEVNEEGTEAVAATVATVILCSSKTSDAPHVDFVADHPFGFFVIEEVSGATLFAGHVLDPTTN

>TraesCS4B02G310200

MQSVKELVLRCLWLCTEAEGEPDHALAASDGLQAFAPAASDGLQAFALVLNKRLADDDAGRSGNLVTSPLSVYAALALVAAGAREGTLDELLRVLGAPSRDFLAGHVRALGEHALADGSRTGGPRVSLACSVWHDVTMPLRPAYRATAAESYKAVARAVNFRQKPEEAREQINAWVAAATNDLIPSILSPDALSSLTVLVLANAIYFKGMWEKPFDKELTKDDKFHCLDGTAVDAPFVRGLGWHDIACHDGFKVLQLRYVQGHSSQGQPQPPPIYSMCVFLPDARDGLWELTDKIASNPDFVRKHLPCGDVMVSDFRLPKFKVNFGMTMEGILQDMGLNEAFEPGKADLSDMAEDGAGKLALEKIIHRAVIEVNEEGTEAAAATVETMTLCSWETSDAPHVDFVADHPFGFFVIEEVSGATLFAGHVLDPTTN

>TraesCS4D02G078000

MATTDIRLSIGHQTRFALRLASAISSPSHAKGSAGNAAFSPLSLHVALSLVAAGAGGATRDQLAAALGSAEKGEAEGLHALAEQVVQVVLADASGAGGPRVAFANGVFVDASLKLKPSFKDLTVGKYKAETQSVDFQTKAAEVAGQVNSWVEKITTGLIKEILPAGSVDNTTRLVLGNALYFKGAWTEKFDASKTKDEKFHLLDGSSVQTPFMSSTKKQYISSSDSLKVLKLPYQQGEDKRQFSMYILLPEAQDGLWNLANKLSTEPEFLENHIPMQKVPVGQFKLPKFKISFGFEASDMLKGLGLQLPFSAEADLSEMVDSPAGLYVSSVFHKSFVEVNEEGTEAAAATASVVTLRSLPVEPVKVDFVADHPFLFLIREDLTGVVLFVGHVFNPLVSA

>TraesCS4D02G090600

MGFRAAETTMRRDPPQSAGSGGLAALSASLARGIAGSPVDSNLVFSPLSIYTALALVAAGARGATLDEILRVLGARSRGELDEFVARAVGGALRDRTDSGGPRVAFACGVWSDLACPLKPGYLKAVVDGGYRAEASTVDFRGDPDSPRQLINAWAARATSNLVDSVLGPGSVTESTRVVLGNAVYFKGKWDQPFDERNTADAPFRRLGGAHPVDVPFMQSWDEQFVAVHDGFKVLKLRYKMADAFLTRDPKKPAPFFPQRAPLLRPDRFSNAASPSRHGSYGRAGPYPCSYGDINTSSTASPNNNMNNFTSNSTQFSLCIFLPGADDGLRSMVDAIASRPGFLHDHLPRRKVEVGEFRVPRFKLSFHDSVVDVLKELGLSLPFSPLGDLSDMTGPDDSGFGMVVDEVVHKAVIEVNEEGTEAVAVTMVTDRYGCAARCSPPRRVDFVADHPFAYFIVEEETGAVVFAGHVVDPSKES

>TraesCS4D02G106100

MRHFSSLPRVLRRSAAPKPNRRSPAPKPHPPSPSPGPVPDAQAPVAPAPMPTRPWEEALDAAQRAFCLPLAGRVLAAAGTGNAAVSPVGVHAALSLASSGARGATRRQLLGTLGCGGGGKGAAADAANVASRVVKRVLKDRAKSGGPQLAFASGVWADASTTLSPEFVETAGGLYCSAAKTVDFKSTPEDAAEQINSWVNKSTRQTITSLLPDGLVDQNTGLVLGSALYFKGRWLDKTDIGKTAEQKFYCLDGTHVLVPFVEYDRTRLFAAHDGFKVIKLPYKQGNNERKFSMYIFLPDAHDGLFELTKKIFSEPAFLEQHLPTEKRHVGIGVPKFTISFQIDMKDFLKDMTLELPFRRDADFKDMVKEGDSKEPLFLSDVLHKVILEVNDDEIEETSVEKSIGKPLPTEHFAADHPFFFLIREEVSATVIFMGHVLDPSSQY

>TraesCS4D02G231200

MATEAMAGAVRDLAALSMRFLLHLGGNQTNLAFSPLSFHYVLVLLAAGATGDTLNQIVSFLGPSGGMAHASLASHAASAFLARGNGSEPDVRCGVGLWVDSSLRLRPAFADMVASRYNATAQAMPFQEKPDEARVEINRWFEDRTGGLIKELMPEGHLDSDTALVIGNALYLRGSWLRPFDREDTVDGDFFLADGSSVRVPFMTSGIRQRISSHPGFKVLQLPYDSGRVGGRHSFSMHIYLPDERDGLQALIRELSSDTGGFLNRSAPAQAVEVGYFKIPKFKVSRKVEASDLLKDMGLERPFCFSHDFAEMVDYSEPLAVRSVLHECVVEVDEDGTMAAAATEAHIMTGCSIGWEEPVRVDFVADHPFLFLIREDESGIVLFAGQVVNPEL

>TraesCS4D02G308100

MQSAKELVLRCLWLCTEAEAEPDHALAASDGLQAFAPAASEGLQAFALGLNKRLADDDAGRSGNLVTSPLSVYAALALVAAGAREGTLDELLRVLGAPSRDFLAGHVRALAEHALADGSRTGGPRVSFACSVWHDVTMPLRPAYRATAAESYKAVACAVNFLQKPEEAREQINAWVAAATNDLIPSILSPDALSSRTVLVLANAIYFKGMWAKPFDKELTKDDKFHRLDGTAVDAPFVRGLGWHDIACHDGFKVLQLRYVQGHSSQGQPPIYSMCVFLPDARDGLWELTDKIACNPDFVRKHLPCGDVMVSDFRLPKFKVNFGMKMEGILQDMGLNEAFEPGKADLSDMAEDGARKLALEKVIHRAVIEVNEEGTEAAAATVETVTLCSWKTSDAPHVDFVADHPFGFFVIEEVSGATLFAGHVLDPTTN

>TraesCS4D02G318300

MATTDSIKLSAAQQTSFALRLASAISSPSNTDSARGNVAFSPLSLHVALSLIAAGAGGATRDQLVATLGAGEAEGLHAFAEQVVQLVLADSSGAGGPRVWFANAVFSDASLPLKPSFKEVAVGKYKAEAHSVDFKTKAAEVAGQVNSWVDKVTSGLIKEILPAGSIETDTRLVLGNALYFKGTWAQKFHASDTKDDNFHLLDKTYFFQKHKTIKAPFMSSTKNQYISCFENLKVLKLPYQQGGDKRQFSMYILLPKAQDGLWSLAGKLTSEPEFLEKHIPARAVPVGQFKIPKFKISFDFEASKLFKSLGLQLPFSTEADLSEMVDSPMGQSLCVSSIFHKSFVEVNEEGTEAAAATIAVVMCRSLPAVPPMKVDFVADHPFLFVIREDVTGVVLFVGHVVNPLLAG

>TraesCS5A02G305500

MARGRGFTGSDALTALTHRLADQLSVTRENPSNVAFSPLSIYSALSLVAAGARGGTLDELLAVLGASSRDELAANGRFVVEHALADRSPSGGPRVAFASGVWHDAGRALEPAYREAVVASYLAEIRAVDFRNKAEESREEINKWVAAATGKLIDSILPPESVSEDTAVVLANAIYFKGKWETPFKKKNTKVERFYLLDGTAVDAPMMRTGRSQYVEEHDGFKVLRLPYGSQDPGASKKRPRSRQSGTSSRDNPAPLPRYSMCVFLPDARDGLWDLVGKIASSPSFLRDHLPEYEVDVDEFRLPKFKVSFYGKLSCVLRDMGLVAAFKADKADLTDMAPDVEDASGELIKRLVLKDVFHRAVVEVNEEGTEAAAVTVCEEEDESACQPVDFIADHPFAFFVIEEVSGAVVFAGHVLDPTKHQDTLHDVD

>TraesCS5A02G323600

MEEEQQSAGMADLAARLTKRLADANRDRNLVFSPLSIYAVLALLAVGASGETLEEILRVLGHPSRRELEDYVERLLDGSLDPDGGGPTVAFACGVWSDLRRPLKPAFRDAVVGRYKAEASSVDFRNDPELARAQINAWAAASTRNLIDSAVPRGTVHRNTHLVLANAIYFRGKWEVPFYKSSTKDRPFHRHDGTAVDVPFMTNHRKYYHYMAVHDGFKVLKLPYESSTAYTQRHCMCIFLPDARDGLASLLDKITSSSPAGFLREHLPTRRVKVDQVLVPRFELSFRSSVTALLKDLGLRLPFSHRADLSEMLNDGYEFRVQDVFQKAVIEVNEDGTEAAALTFYDVTAKSSQYPPEEVLFVADHPFAYFIVEEESHAILFAGHVVDPSDATGVVIPSGERSTTKGKIDKVDTESGRKRRRDEQPQSSHGHSSSRQHRRDESRRAASSEYRREESRYYRSERRERTNNPRREYNTKIQFC

>TraesCS5A02G359700

MATTLATDVRLSIAHQTRFALRLASTISSNPKSAASNAVFSPVSLHVALSLLAAGAGSATRDQLVATLGTGEVEGLHALAEQVVQFVLADASSAGGPHVAFANGVFVDASLPLKPSFQELAVCKYKADTQSVDFQTKAAEVATQVNSWVEKVTSGRIKDILPSGSVDNTTKLVLANALYFKGAWTDQFDSSGTKNDYFYLPDGSSVQTPFMSSMDDQYLSSSDGLKVLKLPYKQGGDKRQFSMYILLPEAPGGLSNLAEKLSAEPDFLERHIPRQRVALRQFKLPKFKISFETEASDLLKCLGLQLPFSNEADFSEMVDSPMAHGLRVSSVFHQAFVEVNEQGTEAAASTAIKMALLQARPPSVMDFIADHPFLFLLREDISGVVLFMGHVVNPLLSS

>TraesCS5A02G410600

MRLVSKHALAADDPSGPLVVTSACSVWCHKDLALKPAYRKAAVKSYKADVRAVDFVKKVIYLWLSISSFNADPSLSCMQPEDARKEINRWVSKATKKLITNVLPRGSVHRDTRLVLTNAIYFKGMWENAFSKSRTKDHTFHRGTASTAAPFRCHSWTETDATSTWSLLKLPYKKAANNGATYSMCVFLPTARDGLRSLADEMATGGPSFLFDHLPTQSRSVTKLRLPKFKLSFFCSMKKVLEMLGLRAAFSGEADLSDMVDKDSAGNDVPLRVEDVFHRAVVEVNEEGTEAAAFTAVMTVLYCWTPPSVPVDFVADHPFAFYIVEEVSHTVVFAGHVLDPSETE

>TraesCS5A02G417800

MATTLATDVRLSIAHQTRFAFRLASAISSNPESTINNAAFSPVSLHVALSLITAGAGGATRNQLATTLGEGEVEGLHALAEQVVQFVLADASSVGGPRVAFANGVFVDASLQLKPSFQELAVCKYKAEAQSVDFQTKAAEVTAQVNSWVEKVTTGLIKDILPAGSIDNNTRLVLGNALYFKGAWTDQFDPRGTEIDYFYLLDGSSIQTPFMYSSEKQYISSSDGLKVLKLPYKQGGDKRQFSMYILLPEARSGIWSLAEKLSAEPEFLEQHIPRQKVALRQFKLPKFKISFGIEASDLLKGLGLQLPFGAEADLSEMVDSPMAQNLYISSIFHKAFVEVNETGTEAAATTIAKAVLRQARPPSVLDFIADHPFLFLIREDTSGVVLFIGHVVNPLLSS

>TraesCS5A02G471300

MSPIGRFVLFLTLFAAWRLRSTLFAEAPPAPGVDSASAIGNASCVALAREAGVRAEGGSGRNFVISPLSIHAALAMVAAGARGDTLRELLGFLGSSSLDALHRTAATELVGRLNGIAQTSFASGVWVDRRWALRPEFTGTIASWYNATAESVDFVSGAEQARQRVNAFVADATNKQILEVLPPGSVNSSTAVVLANALYFKGAWTQPFDVSTAPFHIPGGTTVHVPSMTTSESQHIAVYPGFRALKLPYKNGVQQHAEFYMLILLPDRDTVSLADLYDKAVSTPEFFKTHTPTGKVPVGRFMVPKFNFTSEFEASSDMRKHGVTRAFESGGFSGMVTGGDKGLAITTVHHKATVEVDEVGTVAAAATAVVMGFGSAGPRAPQDLVDFVADRPFLFAVVEEGTDAVLFLGHVANPLAH

>TraesCS5A02G471430

MSPFGKFLFCLTLLAAWRLSSTLFTEAPPAPGVDPAFRNASCLALAREAGVRAEGGTGSNFVISPLSIHAALAKVAADARGDMLDELLQFLGSASLNELHRAMATELVGRLNSIAQTSFASGVLVDRMLALKPKFTAIAASRYNAMVESVDFVSGAEQARQRVNAFVVDATNKQILQVLPPGSINSGTAVILANALYFKGAWTQPFDVSNAPFHIPGGTTVRVPSMTTSESQQIEVYPGFRALKLSYKNDVQQQAEFYMPILLPDSETEIVDLYDKAVSTLEFIKTHTLTKKVPVGQFMVPKFKFTSEFEVSSDMRKLGVTRAFRGSDFSGMMTGGEGISITGVYHKATIKVDEVGTVAAAATAVLCFGSAAPGAPRDLVDFVADRPFLFAVVEEGTDAVLFLGHLANPLAH

>TraesCS5A02G471500

MSPFGKFLFCLTLLAAWRLSSTLFTEAPPAPGVDPAFRNASCLALAREAGVRAEGGTGSNFVISPLSIHAAFAKVAAGARGDTLNELLRFLGSASLNELHRAAATELVGRLNGIAQTSFASGVWVDRMLALKPEFTAIAASRYNATVESVDFVSGAEQARQRVNAFVADATNKQILQVLPPGSVNSGTAVVLANALYFKGAWTQPFDVSTAPFHIPGGTTVRVPSMTTSESQQIAVYPGFRALKLPYKNDAVSTPEFIKTHTPTKKVPVGQFMVPKFKFTSEFEVSSAMRKLGVTRAFQGGDFSSMMTGGEGISITGVYHKATIEVDEVGTVAAAATAVLYFGSAAPGAPRDLVDFVADRPFLFAMVEEGIDAVLFLGHLANPLAH

>TraesCS5A02G490400

MATTDSIRLSAAQQTSFALRLASAVSSPSNADGARGNVAFSPLSLHVALSLIAAGASGATHDQLVATLGAGEAEGLHAFAEQVVRLVLADSSGAGGPRVSFANAVFSDASLPLKPSFKEVAVGKYRAETHSVDFKTKAAEVAGQVNLWVDQVTSGLIKEILPAGSIEADTRLVLGNALYFKGTWVQKFHASDTKDDNFYLLDKTYFFQKHKTIKAPFMSSTKNQYISSFENLKVLKLPYQHGGDKTQFSMYILLPKAQDGLWSLARKLTSEPEFLEKHIPARAVPVGQFRIPKFKISFDFEASKLFKSLGLQLPFSTEADLSEMVDSPLGQSLCISSIFHKSFVEVNEEGTEAAAATFAVAMSRSLSAVPPRKVDFVADHPFLFVIREDVTGVVLFVGHVVNPMLAA

>TraesCS5B02G306000

MARGFTGSDALTALTHRLADQLSVTRETPSNVAFSPLTIYSALSLVAAGARGSTLDELLAVLGASSRDELAANGRFVAERALADRSPSGGPRVVFASGVWHDAGRPLEPAYREAVAASYLAEIRAVDFRNKADESREEINKWVAAATGNLIDSILPAESVGEDTAVVLANAIYFKGKWETPFKKKKTKVERFYLLDGTAVDAPMMRTGRSQYIDEHDGFKVLRLPYRSKDPGASKKRRRGTSSGDDPAPPLPRYSMCVFLPDARDGLWDLVGKIASSPSFLRDHLPEYEVDVDEFRLPKFKVSFYGKLSGVLGDMGLVATFKADKADLTGMAPDVEDTSGDIKRLVLKDVFQRAVVEVNEEGTEAAAVTVCEEEDESACQPVDFIADHPFAFFVIEEVSGAVVFAGHVLDPTKHQDTLHDVD

>TraesCS5B02G324100

MEEQQSAGMADLAARLTKRLADANSDRNLVFSPLSIYAALALLAVGAGGETLEEILRVLGARSRRELEDSVERLLDGSLDPDGGGPSVAFACGVWSDLRRPLKPAFRDAVVGRYKAEASSVDFRNDPEQARAKINAWAAASTRNLIDSAVPRGAVHRNTHLVLANAIYFRGKWEMPFYKSSTKDRPFHRHDGFKVLKLPYESSTAYTQRHSMCVFLPDARDGLAGLLDKITTSSPAGFLREHLPTRRVKVDQVLVPRFELSFRSSVTAVLKDLGLRLPFSHRADLSEMLDDGYEFCVQDVFQKAVIEVNEDGTKAAALTFYDVTAKSSQYPPEEVLFVADHPFAYFIIEEESHAILFAGHVVDPSDCTGAVIPSGERNTTKGKIDKVDAESGRKRPRDEQPQSSHGHSSSRQHRRDESRRVASSEYRGEESRYYRSERRERTDYPRREHNSRYY

>TraesCS5B02G362000*

MATTLATDVRLSIAHQTRFALRLASTISSNPKSAASNAAFSPVSLYSALSLLAAGAGSATRDQLVATLGTGKVEGLHALAEQVVQFVLADASSTGGPRVAFANGVFVDASLLLKPSFQEIAVCKYKAETQSVDFQTKAAEVTTQVNSWVEKVTSGRIKDILPPGSIDNTTKLVLANALYFKGAWTEQFDSYGTKNDYFYLLDGSSVQTPFMSSMDDQYLLSSDGLKVLKLPYKQGGDNRQFFMYILLPEAPGGLSSLAEKLSAEPDFLERHIPRQRVALRQFKLPKFKISFGIEASDLLKCLGLQLPFGDEADFSEMVDSLMPQGLRVSSVFHQAFVEVNEQGTEAAASTAIKMVLQQARPPSVMDFIADHPFLFLVREDISGVVLFMGHVVNPLLSS

>TraesCS5B02G402400

MDATRDTTQSCSGGQAALAASLASRLADDNADSNLVLSPLSIYAALALLAAGARGATLDEILGVLGAPSRAALDEFLSRVAEDALKDHSESGGPRVAFAYGVWTDLACPLKPAYRHAAVSTYKADASTVDFRNNPEAARSQINAWVAQVTSNLIRSVLRPGSITPLTRVVLGNAIYFKGKWEKPFDKEDTANKPFHRLDGRTVDVPFMKSWSSQYIAVHDGFKVLKLRYRMAQAQGNPFVHSQSRSFHSRFLHKHLPKEKIDVRKFRVPKFKLSFHNSLVTVLKKLGLQLPFSDQADLSDMVEMPFVLSDVIHKAVIEVNEEGTEAAASTLMHIGAGCCAMPPSPPPPVDFVADHPFAYFIVEEATGIVVFAGHVVDPSNKD

>TraesCS5B02G414600

MEKAARRPSKKARRTPADSDGLTALALRLSKKFSGREEYEGQNIMFSPLSIYTALGLLAAGAQGDTLDEILAVLGATSRDEVAAVMRLVSKHALAADDPSGPLVVTSACSVWCHKDLPLKPAYRKAAVKSYKADARAVDFVRKPEDARKEINRWVAKATKKLITSVLPRGSVHGHTRLVLTNAIYIKGKWENAFSKSRTKDDTFHRLDGSTVRVPFMDGNGRDKYLVSTFDGFKVLKLPYKKAANNGATYSMCVFLPTARDGLRSLADEMATGGPSFLFDHLPTQSRSVTKLRLPKFKLSFFCSMKKVLESLGLRAAFSGEADLSDMVDKDSAGNDVPLRVEDVFHRAVVEVNEEGTEAAAFTAVMTVLYCWTPPSVPVDFVADHPFAFYIVEEVSRAVVFAGHVLDPSETE

>TraesCS5B02G419900

MATTLATDVRLSIAHQTRFGFRLASTISSNPESTANNVAFSPVSLHVALSLITAGAGGATRDQLVATLGEGEAERLHALAEQVVQFVLADASYADSPRVTFANGVFVDASLPLKPSFQELAVCKYKAEAQSVDFQTKAAEVTAQVNSWVEKVTTGLIKDILPAGSISNTTRLVLGNALYFKGAWTDQFDSRVTKSDYFYLLDGSSIQTPFMYSSEEQYISSSDGLKVLKLPYKQGGDKRQFSMYILLPEAPSGIWSLAEKLSAEPELLERHIPRQKVALRQFKLPKFKISFGIEASDLLKHLGLQLPFSDEADLSEMVDSPMPQGLRISSVFHKTFVEVNETGTEAAAATIAKAVLLSASPPSDMDFIADHPFLFLIREDTSGVVLFIGHVVNPLRSL

>TraesCS5B02G461200

MDPATTMPRPRKKSRQSGSARVGQPKQRDALMEQSLPATGYELPVTKNPSVQLDVVGPTGNPGSSGLAALAVGLARRLADGSVDDNLVFSPLSIYTALALLAAGARDATLDEILRVLGARSRSELENFVSHMAADALQDRSASGGPRIAFACGIWSDLTRCLKLSFREAVVGTYKAEASSVDFRGTPEAARSQINGWAAQVTRNLIDSVLPAGSISPATRVVLGNAMYFKGNWEDQPFDKRHTAHEPFYRLDRSQVDVPFMQSSDSQFVAVHDGFKVLMLQYKMAAPDYQEQATSNSDHSGHTQFSMCIFLPDAHDGLLGLLDTIASRPGFLQDHLPEVQITLSEFRVPKFKLSFHSSVVAVLKKLGLELPFCLEGDLADMVEDDGSGLPIVVEDVIHKAVVEVNEEGTEAAAVTMVISAPRCARRGRRPPPPQVNFIADHPFAYYIVEEATGAVVFAGHVVDPSKE

>TraesCS5B02G483500

MSPIAKFVFCLTLLAAWRLSSTLFAEAPPAPGVDSASRNASCLALAREAGIRSEGGTGRNFVISPLSIHAALAKVAAGARGDTLSELLRFLGSASLNELHRAAATKLVGRLNGIGQTSFTSGVWVDRMLALKPEFMAIVASRYNATAESVDFVSGAEQARQRVNAFVADATNKQILEVLPPGSVGPGTAVVLANALYFKGAWTQPFDVSTAPFHIPGGTTVRVPSMTTSEPQHIAVYPGFRALKLPYKNDVQQQAEFYMLILLPDSETEIADLYDKAVSMREFIKTHTPTENVPVRQFMVPKFKFTSEFEVSSDMRKLGITRAFEGGDFSGMMTGGEGLSINGVYHKATIEVDEVGTVAAAATAVLWFGSAAPGAPQDLVDFVADRPFLFAVVEEGTDAVLFLGHVANPLTH

>TraesCS5D02G312500

MARRFTGSDALTALTHRLAHQLSVTRETPSNVAFSPLSIYSALSLVAAGARGGTLDELLAVLGASSRDELAANGRFVAEHALADRSPSGGPRVAFASGVWHDAGRALEPAYREAVVASYLAEIRAVDFRNKAEESREEINKWVAAATGKLIDSILPAESVDEDTAVVLASAIYFKGKWETPFRKKRTKVERFYLLDGTAVDAPMMRTGRSQYIDEHDGFKVLRLPYRSQDPGASKKRRRGTSSGDDPAPPLPRYSMCVFLPDARDGLWDLVGKIASSPSFLRDHLPEYEVDVDEFRLPKFKVSFYGKLSGVLGDMGLVAAFKADKADLTGMAPDVEDASGDIKRLVLKNVFHRAVVEVNEEGTEAAAVTVCEEEDESACQPVDFVADHPFAFFVIEEVSGAVVFAGHVLDPTKHQDTLHDVD

>TraesCS5D02G368900

MATTLATDVRLSIAHQTRFALRLASTISSNPKSAASNAAFSPVSLHSALSLLAAGAGSATRDQLVATLGTGEVEGLHALAEQVVQFVLADASSAGGPRVAFANGVFVDASLLLKPSFQELAVCKYKAETQSVDFQTKAAEVTTQVNSWVEKVTSGRIKNILPSGSVDNTTKLVLANALYFKGAWTDQFDSYGTKNDYFYLLDGSSVQTPFMSSMDDDQYISSSDGLKVLKLPYKQGGDNRQFSMYILLPEAPGGLSSLAEKLSAEPDFLERHIPRQRVAIRQFKLPKFKISFGIEASDLLKCLGLQLPFSDEADFSEMVDSPMPQGLRVSSVFHQAFVEVNEQGTEAAASTAIKMVPQQARPPSVMDFIADHPFLFLLREDISGVVLFMGHVVNPLLSS

>TraesCS5D02G425800

MATTLATDVRLSIAHQTRFAFRLASAISSNPESTVNNAAFSPVSLHVALSLITAGAGGATRNQLAATLGEGEVEGLHALAEQVVQFVLADASNIGGPRVAFANGVFVDASLQLKPSFQELAVCKYKAEAQSVDFQTKAAEVTAQVNSWVEKVTTGLIKDILPAGSIDNTTRLVLGNALYFKGAWTDQFDPRATQSDDFYLLDGSSIQTPFMYSSEEQYISSSDGLKVLKLPYKQGGDKRQFSMYILLPEALSGLWSLAEKLSAEPEFLEQHIPRQKVALRQFKLPKFKISLGIEASDLLKGLGLQLPFGAEADLSEMVDSPMAQNLYISSIFHKAFVEVNETGTEAAATTIAKVVLRQAPPPSVLDFIVDHPFLFLIREDTSGVVLFIGHVVNPLLSS

>TraesCS5D02G462700

MKPRSKKSAALMDPATTMARPRQKSRQSGSGLARPRKKSRQSGSARVGQPKQRDALKEQMLPARGYELPVTKNPSVQFDVVGPTGHPGSSSLAALAVGLARRLADGSADDNLVFSPLSIYTALALLAAGARDATLDEILGVLGARSRSELENFVSHMAADALQDRSASGGPCIAFACGIWSDLTRRLKPAFREAVVGTYKAEASTVDFRGAPEAARKQINAWAAQVTRNLIDSVLPAGSISRATQVVLGNAMYFKGKWQDQPFDKRYTAHKPFHRLDHSQVDVPFMQSWKSQFVAVHDGFKFSMCIFLPDAHDGLLGLLDTIASRHGFLQDHLPRQRIALGEFRVPKFKLSFHSSVVAILKKLGLALPFCLEGDLSDMVEDDGSGLPIVVEDVIHKAVVEVNEEGTEAAAATMVRKGIGCAPRGRRPPPPEVDFIADHPFAYYMVEEATGAVVFAGHVLDPSKE

>TraesCS5D02G483800

MSPFGKFLFCLTLFAAWRLSSTLFAEAPPAPGVDSASRNASCVALAREAGVRAEGGTGSNFVMSPLSIHAALAKVAAGARGDTLNELLRFLGSASLNELHRAAATKLVGRLNGIAQTSFASGVWVDRMLALKPEFTAIAASRYNATAESVDFVSEAEHARQRVNAFVADATNKQILEVLPPGSVDSRTAVVLANALYFKGAWTQPFDVSTAPFHIPGGTTVRVPSMTTSESQHIAVYPGFRALKLPYKNDVQQQAEFYMLILLPDRDTVTLADLYDKAVSTPEFIKTHTPTGKVPVGQFMVPKFKFTSKFEASSDMRKLGVTRAFEGGDFSGMMTGGEGLSINGVYHKATIEVDEVGTVAATATAIVEFGSAGPGHGVDFVADRPFLFAVVEEGTDAVLFLGHVANPLAH

>TraesCS6A02G042000

MSRFGKAVLLCLTLFAAWHLCSALFTAAPPGPGADHAEGSHASCLPLAREVGVRAAAGTGSNFVFSPLSIHAALAMVTAGARGDTRRELLRFLGSASLHELHHAPANELVGRLNGLTQTSFACGVWVDRRHALRPEFTATGASRYGATAESVDFVSGAEQARQRVNGFVADATKQLIRDILPPGSVDSSTAVVLANALYFKGAWSHPFDVFTAPFHVPGGTTVGVPSMTTGRSQYIALYPGFRALKLHYKNDVQWQADAFYMLILLPDSGILSLADLYNKAVSTPEFISKHTPVEEVPVGRFMVPKFKFTFEFEASSDMQKLGVTRAFSGGDFSGMVSGEDGLSISRVYHKATIEVDEQGTVAAAATVVVLMEGAALEEREPPHLVDFVADRPFLFAVVEERTDAVLFLGHVVNPLAG

>TraesCS6A02G042100

MSRFGKTVLLCLALFAAWHLCSTLFAGAPPGGHSEEEKAVVSDAAGNASGLSLAREAGVRAAAGTGRNFVVSPLSIHAALAMVAAGARGETRRELLGLLGSASLDELHRAPAIKLVGRLNGLKQTSFACGVWVDRRRALRPEFTATGASRYAATAESVDFVSGAEQARRRVNGFVADATKQRIRDVLPPGSVDSSTTVVLANALYFKGAWPEPFDVFTAPFHTPGGATVRVPSMTTGRSQYVALYPGFRALKLPYRNDGDRSAAFHMLILLPDSGGLSLSDIYDKVVSSPEFIRKHTPEEEVEVRRFMVPKFKFTTEFEASSDMRKLGVTRAFAGGDFSGMVSGGDGRLSIGGVHHKATIEVDEQGTVAAAATATDMAGSALPSEPPHFVDFVADRPFLFAVVEERTGTTLFLGHVVNPLAN

>TraesCS6A02G042200

MSRFGKAVLLCLALFTAWHLCSTLFADAPPAPGGDPAEEAPVASDAAGKASGLPLAREAGVREAAGAERNFVISPLSIHAALAMVTAGARGDTRRELLGFLGSASLDELHRSPAIKLVGRLNGLTQTSFACGVWVDQRRALRPEFTATGASRYAATAESVDFVSGAELARRRVNAFVADATNQRIHDILPPGSVHSSTEVVLANALYFKGAWRRPFDVFTAPFHIPGGTTVRVPSLTTGRSQYIALYPGFRALKLPYRTDGDRQAAAFYMLILLPDSGILSLTDLYDKAVSMPEFIRKHTPEEEVPVGQFMVPKFKFTSKYEASSDMQKLGVTTAFKGGDFSGMVSGGDGRLSIGRVLHKATIEVDELGTVAAAATAIVMYGSALRSEPPHLVDFVADRPFLFAVVEERTGTTLFLGHVVNPLAN

>TraesCS6A02G124400

MSSWRPTGSHGSPPRRGRPHDEHPGTLPAPPHFAGSQAPQMFPAAAVARHLASFQTAATSNTRQQPTDNTEACHPPHHHRPADAASRFTAPWPPTFAPPRPSFPSWAAAVRPTAMIPRETGRTHDVYSGCTAGAGVGNTVTNAGCLRMAASVGSTAAGGGRNFIVSPLSLHAALALVAAGAKGETQRELLDFLVGPAGSSLAALHGDPAIRLVGMLRGLEQTSFACGVWVAHGRALRPEFVEVAGAVYAAVAESVDFRSEPEKARQQVNTFVKHETKELIDEVLPARSVDSSTVVVLANALYFKGTWAQPFDPSATFAAPFHLADGTTVRAPFMTTSLFQQHVAAFPGFKALKLPYKNGGGFHHVHQAASFYMLLLVPDHSAALGLAGLYGKAVSTPDFIRRHTPADQVPVGRFMVPKFRFEFKFEASREMQELGVARAFGGGDFSGMVTGGNGLRISGVYHNATVEVDELGTVAAAATAVCLSQCASARPPVDFVADRPFLFAIVEERTGVALFLGHVVNPLDG

>TraesCS6B02G041700

MEDQRGQTRPRKKARKSKSPPAVGSGGLTAFALRLAKKLAEGDDTRGSNIAFSPLSLYTTLGLVAAGARGTTLDELLALLGASSSHEVAGFVRGLSATEPSGSVGGPLITYAYGVFHQKNMELTPAYLRTAAESYKAEIGAIDFAQEGSREKTRKEINEWAAAATDNLIPEILPEGSLSDGSRLVLTNAIYFKGVWESRFPEALTEHRTFYRLGGASAAIIVPFMTFYPGLHDLFVSYHKDFDVLKLPYKTGDGTARYSMCVFLPHARGGLRAMTNALAARGSLLDHVPKHTIKVTKLLLPKFKLSFFCRLAEVLESLGLRQAFSKEADLSGLVERSVCDVRLDEVFHKAVVEVNEEGTKAVACTAVSGRRKQCARRVAPREFVADHPFAVYIVEEVSGAVVFAGHVLDPSSSSQ

>TraesCS6B02G057800

MSRFGKTVLLCLALFAAWHLCSTLFAGAPPGGHSEEKKAVVSDAAGNASGLSLAREAGVRAAAGTGRNFVVSPLSIHAALAMVAAGARGETRRELLGLLGSASLDELHRAPAIKLVGRLNGLKQTSFACGVWVDRRRALRPEFTATGASRYAATAESVDFVSGAEQARRRVNDFVADATKQRIRHILPPGSVDSSTAVVLANALYFKGTWPEPFDVFTAPFHTPGGATVRVPAMTTGRSQYIALYPGFRALKLPYRNDGDHRAAFYMLILLPDSGTLSLTDLYDMAVSSPEFIRKHTPEEEVEVRRFMVPKFKFTTEFEASSDMRKLGVTSAFAGGDFSGMVSGGDGRLSIGGVHHKATIEVDEQGTVASAATATDMAGSALPSEPPHFVDFVADRPFLFAVVEERTGTTLFLGHVVNPSR

>TraesCS6B02G058100

MSRFEKAVLLCLALFAAWHLCSTLFADAPPRPGGHSAEEKAVVSGAAGNASGLSLAREAGVRAADGAGSNFVISPLSIHAALALVAAGARGVTQTELLGLLGSASLDELHRAPTIKLVGRLNGLTQTSFACGVWVDRRQALRPEFMATGASRYGATAESVDFVLEAEQARRRVNAFVANVTKQLILDVLPPGSVHSSTAVVLANALYFKGAWSQPFDVFTAPFHIPGGSTVGVPSMKTYQSQYIALYPGFRALKLPYKNEVDQQAAFYMLILLPDSYTLSLAYLYDKAASTPEFIKKHTPARKVPVGQFMVPKFKFTFEFEVSSDMKKLGVTRAFNRGNFSGMVSGMDELSITGVYHKATIEVDELGTVAAAATAIVIGTTSVGRSRALMDFVADRPFLFAVVEERTSTVLFLGHVVNPQLG

>TraesCS6B02G152500

MSSWRPAGPHGSPPRRGRRHDDHPGTLPAPPHFPGSQAPQMFPAAAIARHLASFQTAPSNTRQLPTENTEACHPHHHRSADAASRFTAPWPPTFAPPRPSPSWAVPAMVPRDTGTHGVYSGCTAGAGVGNTVTNAGCLRMATGVGSTAAGGGRNFIVSPLSLHAALALVAAGAKGETQRELLDFLMGPAGSSLAALHGDPAIRLVGMLRGLEQTSFACGVWVARGRALRPEFVEVAGAVYAAVAESVDFWSEPEKARQQVNTFVKHETKELIDEVLPVGSVGSSTVVVLANALYFKGTWAQPFDPSATFGAAFHLADGTTVLAPFMTTSLFQQHVAAFPGFKALKLPYKNGGSFHVHQAALFYMLLLVPDHSADLGLAGLYDKAVSTPDFIRRHTPADQAPVGRFMVPKFKFEFKFEASREMQELGVTRAFGGGDFSGMVTGGNGLRISGVYHSATVEVDELGTVAAAATAVCMQQCGSARPPVDFVADRPFLFAIVEERTGVALFLGHVVNPLDG

>TraesCS6D02G048100

MARTHGKPIPISSFGKAVVLCLALFAAWRLCSTLFADAPPRPRFNVRDASCLPLAREVGVRAAAGTGSNFVVSPLSIHAALAMVTAGARGDTRRELLRFLGSASLNELHRAPSNELVGRLNGIAQTSFACGVWVDRRRALRLEFAATGASRYGATAESVDFVSGAEQARLRVNAFVADATKQLIRDILPPGSVDSSTAVVLANALYFKGAWSHPFDLFTAPFHVPGGTTVGVPSMTTGRSQYIALYPGFRALKLPYKNDVLRQADAFYMLILLPDSGTLSLADIYDKAVSTPEFIRKHKPVKKVPVGRFMVPKFKFTFEFEASSDMGKLGVTRAFKVGDFSGMVSSGDGHGQVSISRVYHKAAIEVDEQGTVAVAATVIRMDGSAFEKKEPPHLVDFVADRPFLFAVVEEMTG

>TraesCS6D02G048700

MSRFGKTVLLCLALFAGWHLCSTLFAGAPPGGHSAEEKAVVSDAAGNASGLSLAREAGVRAAAGMGRNFVVSPLSIHAALAMVAAGARGETRRELLGLLGSASLDELHRAPAIKLVGRLNGLKQTSFACGVWVDRRRALRPEFTATGASRYAATAESVDFVSGAEQARRRVNGFVADKTKQRIRHILPPGSVDSSTAVILANALYFKGAWPEPFYVFTAPFHIPGGATVRVPSMTTGRSQYIALYPGFRALKLPYRNDGDHRAAFYMLILLPDSGALSLPDLYDKVVSSPEFIRKHTPEEEVEVRRFMVPRFKFTTEFEASSDMRKLGVTRAFAGGDFSGMVSGGDGRLSIGGVHHKATIEVDEQGTVAAAATAIDMAGSALPSEPPHFVDFVADRPFLFAVVEERTGTTLFLGHVVNPLAN

>TraesCS6D02G048800

MSRFGKAVLLCLALFAAWHFCSPLFADVPPRPGGDPAEEAPVASDAAGKASGLPLAREAGVREAAGAGRNFVISPLSIHAALAMVTAGARGDTRRELLGFLGSASLDELHRSPAIQLVGRLNGLTQTSFACGVWVDQRRALRPEFTATGASRYVATAESVDFVSGAELARRRVNAFVADATNQRIHDILPPGSVHSSTAVVLANALYFKGAWRRPFDVFTAPFHIPGGTTVRVPSMTTGRSQYIALYPGFRALKLPYRTDGDRQAAAFYMLILLPDNATLSLTDLYGMAVSMPEFIRKHTPEEEVPVGQFMVPKFKFTSEYEASSDMQKLGVTTAFKGGDFSGMVSGGDGRLSIGGVLHKATIEVDEQGTMAAAATAIAMYGSALRREPPHLVDFVADRPFLFAVVEERTGTTLFLGHVVNPLAN

>TraesCS6D02G114700

MSFWRPTGSHGSPPRRGRRHDDHPGTLPAPPHFPGSQAPQMFPAAAIARRLASFQTAATSNTRQLPTQNTEACHPPHHHRSADAASRFTVPWPPTFAPPRPSFPSWAVPMRPTAMVPRETGTRGHYSGDTAGAGVGKAATNAGCLRMAASVGSTAAGGGRNFIVSPLSLHAALALVAASAKGETQRELLDFLVGPAGSSLAALHGDPAIRLVGMLRGLEQTSFACGVWVARGRALRPEFVEVAGAVYAAVAESVDFWSEPEKARQQVNTFVKHETKELIDEVLPAGSVDSSTVVVLANALYFKGTWAQPFDPSATFAAPFHLPDGTTVHAPFMTTSLFQQHVAAFPGFQALKLPYGNGGGFHVHQAASFYMLLLVPDHSAALGLAGLYDKAVSTPDFIRRHTPADQVPVGRFMVPKFKFEFKFEASREMQELGVTRAFGGGDFSGMVTGGNGLFISGVYHSATVEVDELGTVAAAATAVCLSQCASARPPVDFVADRPFLFAIVEERTGVALFLGHVVNPLDG

>TraesCS7B02G006500

MQFLKPRTVRLYPKLRRWILTDAASSGPGLQPLALALNKRLADDAGKSNKNLVFSPLSICAALSLVAAGARERTLAEMLDVLGARSRDDLAGSVRALAEQALADQSWAGGPHVSFACAVWHDKTRPLKPGYADAAVKSYKAQTCAVDFHEKPKEAGEQISAWVAASTNKLIPSIVDPDTLSNQTDLVVANAIYFKGKWDKPFDEEDTKEDKFHRRDSSTIDVPFMRGWGKQRIACHEGFKVLQLRYQRGLPSPTQPPAHYSMCIFLPDDRDGLSQLTDRMAGDPDFLREHLPTSTVLVGDFRLPKFKLAFDTELAGVFHDLGLKDAFDPEKADFTDMAEGAGRPLALEEMLHKAVIEVNEEGTEAAAVTAALMFGCASGYPPPCVDFVADHPFAFFVMEEASGAIMFAGHVLDPSSE

>TraesCS7D02G103400

MQFLKPRTVRLYPKLRRWILTDAASSGSGLQPLALALNKRLADDAGKSNKNLVFSPLSIYAALSLVAAGARERTLAEMLGVLGARSRDDLAGSVRALAEQALADQSWAGGPHVSFACAVWHDKTRPLKPDYVAAAVKSYKAQTCAVDFHEKPKEAAEQINAWVAASTNKLIPSIVDPDTLSNQTDLVVANAIYFKGKWDKPFDEEDTKEDKFHRRDSSTIDVPFMRGWGKQRIACHDGFKVLQLRYRRGLSSPARYSMCIFLPDDRDGLSQLSDRIAADPDFLREHLPTSTVLVGDFRLPKFKLAFDTELTGVLQDLGLKDAFDPGKADFTDMAEGTFRPLALEEVLHKAVIEVNEEGTEAAAVTAALMFGCASDYPPQCVDFVADHPFAFFVMEEASGAIMFAGHVLDPSS

>TraesCS7D02G172000

MATTLATDVCLSVAHQTRFALRLASAISSDPESATGNVAFSPVSLHVALSLITAGAGGTTRDQLVAILGNENAGGPEGLHSLAEQVVQLVLADASITGDPRVAFANGVFVDASLSLKPSFQELAVCNYKSEVQSVDFQNKAPEIASQVNSWVENVTTGLIREILPEGSIDYTTRLVLGNALYFKGLWTEKFDESKTKYDKFHLLNGNTVQTPFMSSTNKQYISSSDGLKVLKLPYQKGGDNRQFSMYILLPERRDDLWTLAKRLSTESEFIEKHIPTEKVVVDQFMLPKFKISFGFEATNLLKSLGLQLPFSREANLSEMVNSQVDLFLSSVFHKSFVEVNEQGTEAAAATSVAIEQQQMPIVMDFVADHPFLFLIREDVTGVVLFIGHVANPLVSS

Note: Gene names ending with * indicate the sequence is manually annotated or corrected.
