# Supplementary material for: Genes Encoding Structurally Conserved Serpins in the Wheat Genome: Identification and Expression Profiles during Plant Development and Abiotic and Biotic Stress
Source: Int J Mol Sci. 2023 Jan 31;24(3):2707. doi: 10.3390/ijms24032707 (PMC9917288; doi:10.3390/ijms24032707)
Supplement: Supplementary file 1 [file ijms-24-02707-s001.zip › File S2_Annotation of WSZ13-2B -2D.docx.docx]

**Supplementary File S2**. Annotation of homeologues of TraesCS2A02G036700 on chromosomes 2B and 2D.

Using the TraesCS2A02G036700 sequence, we searched the wheat TGACv1 scaffold and found the homologous sequence on chromosome 2B, the protein product of which was named WSZ13-B. This sequence was then confirmed in IWGSC RefSeq v1.0. The blast result is below.

>WSZ13-B-CDS on chr2B [
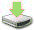
](https://urgi.versailles.inrae.fr/download/iwgsc/IWGSC_RefSeq_Assemblies/v1.0/) [
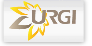
](http://wheat-urgi.versailles.inrae.fr/Seq-Repository/Assemblies)

Length=801256715

Score = 1508 bits (1672), Expect = 0.0

Identities = 836/836 (100%), Gaps = 0/836 (0%)

Strand=Plus/Plus

Query 458 AGCCGGAGGAGGCTAGGGAGGAGATCAACGCATGGGTATCGGCGTCGACGGACGGCCTCA 517

||||||||||||||||||||||||||||||||||||||||||||||||||||||||||||

Sbjct 24889726 AGCCGGAGGAGGCTAGGGAGGAGATCAACGCATGGGTATCGGCGTCGACGGACGGCCTCA 24889785

Query 518 TCCCCTCCATCCTTAGCAGGGGTGCGCTGTCAGACCTCACCGACCTGGTGCTCGCCAACG 577

||||||||||||||||||||||||||||||||||||||||||||||||||||||||||||

Sbjct 24889786 TCCCCTCCATCCTTAGCAGGGGTGCGCTGTCAGACCTCACCGACCTGGTGCTCGCCAACG 24889845

Query 578 CCATCTACTTCAAGGGCAAGTGGGCGAAGCCATTCGCCGGGTATCTCACCCAGGACGACA 637

||||||||||||||||||||||||||||||||||||||||||||||||||||||||||||

Sbjct 24889846 CCATCTACTTCAAGGGCAAGTGGGCGAAGCCATTCGCCGGGTATCTCACCCAGGACGACA 24889905

Query 638 AGTTCCACCGCCTCGACGGCACCGCCGTCGATGCCCCCTTCATGTGCGGTCAAGGCAGGC 697

||||||||||||||||||||||||||||||||||||||||||||||||||||||||||||

Sbjct 24889906 AGTTCCACCGCCTCGACGGCACCGCCGTCGATGCCCCCTTCATGTGCGGTCAAGGCAGGC 24889965

Query 698 ACAACATCGCTTGCCACCATGGCTTCAAGGTGCTCCAGCTCCGCTACGAGGAAGGGCACG 757

||||||||||||||||||||||||||||||||||||||||||||||||||||||||||||

Sbjct 24889966 ACAACATCGCTTGCCACCATGGCTTCAAGGTGCTCCAGCTCCGCTACGAGGAAGGGCACG 24890025

Query 758 GCCCCTTGCTGCCCCAGCAGCCGGCTCTAGCTCCGACGGCGGCGCCGATCTACTCGATGT 817

||||||||||||||||||||||||||||||||||||||||||||||||||||||||||||

Sbjct 24890026 GCCCCTTGCTGCCCCAGCAGCCGGCTCTAGCTCCGACGGCGGCGCCGATCTACTCGATGT 24890085

Query 818 GCGTCTTCCTCCCGGATGCGCGCCGCGGGCTTTGGCGGCTCACCGACAAGATCGCGTGCA 877

||||||||||||||||||||||||||||||||||||||||||||||||||||||||||||

Sbjct 24890086 GCGTCTTCCTCCCGGATGCGCGCCGCGGGCTTTGGCGGCTCACCGACAAGATCGCGTGCA 24890145

Query 878 ACCCCGACTTTCTGCGCAAGCACCTGCCGAGGGACACTGTCCTGGTCGGCGACTTCCGGT 937

||||||||||||||||||||||||||||||||||||||||||||||||||||||||||||

Sbjct 24890146 ACCCCGACTTTCTGCGCAAGCACCTGCCGAGGGACACTGTCCTGGTCGGCGACTTCCGGT 24890205

Query 938 TGCCTAAATTCAAAGTCACCTTCGACATGACGATGAACGACGTTCTCCAGGAGATGGGGG 997

||||||||||||||||||||||||||||||||||||||||||||||||||||||||||||

Sbjct 24890206 TGCCTAAATTCAAAGTCACCTTCGACATGACGATGAACGACGTTCTCCAGGAGATGGGGG 24890265

Query 998 TCAAGGAGGCGTTCGAGCTGGGGAAGGCCGACCTGTCCGACATGGCGGAGGACGGCGCAA 1057

||||||||||||||||||||||||||||||||||||||||||||||||||||||||||||

Sbjct 24890266 TCAAGGAGGCGTTCGAGCTGGGGAAGGCCGACCTGTCCGACATGGCGGAGGACGGCGCAA 24890325

Query 1058 GGAGGAAGCTGGCACTAGAGCAGGTGATCCACAGGGCCGTCATCGAGGTGAACGAGGAAG 1117

||||||||||||||||||||||||||||||||||||||||||||||||||||||||||||

Sbjct 24890326 GGAGGAAGCTGGCACTAGAGCAGGTGATCCACAGGGCCGTCATCGAGGTGAACGAGGAAG 24890385

Query 1118 GCACCGAGGCGGCGGCCGCCACTTGCATGACGCGCCTTGGGTGCACACCGCACTCGCTGC 1177

||||||||||||||||||||||||||||||||||||||||||||||||||||||||||||

Sbjct 24890386 GCACCGAGGCGGCGGCCGCCACTTGCATGACGCGCCTTGGGTGCACACCGCACTCGCTGC 24890445

Query 1178 CGCTTGCACCGTGTGTGGACTTCGTGGCTGACCATCCATTTGCCTTCTTCATCGTCGAGG 1237

||||||||||||||||||||||||||||||||||||||||||||||||||||||||||||

Sbjct 24890446 CGCTTGCACCGTGTGTGGACTTCGTGGCTGACCATCCATTTGCCTTCTTCATCGTCGAGG 24890505

Query 1238 AGGTGTCAGGCGCGATTCTGTTTGCGGGGCACGTCCTTGATCCCACCATCAAGTGA 1293

||||||||||||||||||||||||||||||||||||||||||||||||||||||||

Sbjct 24890506 AGGTGTCAGGCGCGATTCTGTTTGCGGGGCACGTCCTTGATCCCACCATCAAGTGA 24890561

Score = 829 bits (918), Expect = 0.0

Identities = 459/459 (100%), Gaps = 0/459 (0%)

Strand=Plus/Plus

Query 1 ATGGAATCTGTGAAGGAGTTCGCTCTCCGCCGCTTCCCAAAGCTTGGCCAATGGTTCTAC 60

||||||||||||||||||||||||||||||||||||||||||||||||||||||||||||

Sbjct 24889172 ATGGAATCTGTGAAGGAGTTCGCTCTCCGCCGCTTCCCAAAGCTTGGCCAATGGTTCTAC 24889231

Query 61 ACCGAGGCCGATGCCGATCAGGCCGCTCCCGGCGATGGCCTGCAGGCTTTCTCCCTCGGC 120

||||||||||||||||||||||||||||||||||||||||||||||||||||||||||||

Sbjct 24889232 ACCGAGGCCGATGCCGATCAGGCCGCTCCCGGCGATGGCCTGCAGGCTTTCTCCCTCGGC 24889291

Query 121 CTGAATAAGCGCCTCGCGCACGACGCCGGCAGGAGGAGCAACCTGGTCTTCTCGCCGCTG 180

||||||||||||||||||||||||||||||||||||||||||||||||||||||||||||

Sbjct 24889292 CTGAATAAGCGCCTCGCGCACGACGCCGGCAGGAGGAGCAACCTGGTCTTCTCGCCGCTG 24889351

Query 181 TCCGTCTACGCCGGGCTCTCGCTGGTAGCCGCGGGCGCCCGCGACCGCACCCTGGACGAG 240

||||||||||||||||||||||||||||||||||||||||||||||||||||||||||||

Sbjct 24889352 TCCGTCTACGCCGGGCTCTCGCTGGTAGCCGCGGGCGCCCGCGACCGCACCCTGGACGAG 24889411

Query 241 CTGCTCGCTGTCCTCGGCGCGCCCTCCCGGGACTTCCTCGCCGGCCACGTCCGCGCGCTG 300

||||||||||||||||||||||||||||||||||||||||||||||||||||||||||||

Sbjct 24889412 CTGCTCGCTGTCCTCGGCGCGCCCTCCCGGGACTTCCTCGCCGGCCACGTCCGCGCGCTG 24889471

Query 301 GCCGAGCAGGCTCTCACCGACCAGTCCAAGACCGGCGGCCCGCGCGTCAGCTTCGCCTGC 360

||||||||||||||||||||||||||||||||||||||||||||||||||||||||||||

Sbjct 24889472 GCCGAGCAGGCTCTCACCGACCAGTCCAAGACCGGCGGCCCGCGCGTCAGCTTCGCCTGC 24889531

Query 361 GGCGTGTGGCATGACCGGACCATGCCCATCCGCCCCGCCTACCGCGATGCCGCCGAGTCC 420

||||||||||||||||||||||||||||||||||||||||||||||||||||||||||||

Sbjct 24889532 GGCGTGTGGCATGACCGGACCATGCCCATCCGCCCCGCCTACCGCGATGCCGCCGAGTCC 24889591

Query 421 TTCAAGGCCGTCGCCCGCGCCGTCAACTTCCGCCAAAAG 459

|||||||||||||||||||||||||||||||||||||||

Sbjct 24889592 TTCAAGGCCGTCGCCCGCGCCGTCAACTTCCGCCAAAAG 24889630

In refseq v1.0, the gene encoding WSZ13-B is on 2B: 24889172.. 24889630, 24889728..24890561; between genes TraesCS2B02G050200 and TraesCS2B02G050300. We named it TraesCS2B02G050210. The TraesCS2B02G050210 cDNA sequence and the translated protein sequence are below.

> TraesCS2B02G050210-CDS

ATGGAATCTGTGAAGGAGTTCGCTCTCCGCCGCTTCCCAAAGCTTGGCCAATGGTTCTACACCGAGGCCGATGCCGATCAGGCCGCTCCCGGCGATGGCCTGCAGGCTTTCTCCCTCGGCCTGAATAAGCGCCTCGCGCACGACGCCGGCAGGAGGAGCAACCTGGTCTTCTCGCCGCTGTCCGTCTACGCCGGGCTCTCGCTGGTAGCCGCGGGCGCCCGCGACCGCACCCTGGACGAGCTGCTCGCTGTCCTCGGCGCGCCCTCCCGGGACTTCCTCGCCGGCCACGTCCGCGCGCTGGCCGAGCAGGCTCTCACCGACCAGTCCAAGACCGGCGGCCCGCGCGTCAGCTTCGCCTGCGGCGTGTGGCATGACCGGACCATGCCCATCCGCCCCGCCTACCGCGATGCCGCCGAGTCCTTCAAGGCCGTCGCCCGCGCCGTCAACTTCCGCCAAAAGCCGGAGGAGGCTAGGGAGGAGATCAACGCATGGGTATCGGCGTCGACGGACGGCCTCATCCCCTCCATCCTTAGCAGGGGTGCGCTGTCAGACCTCACCGACCTGGTGCTCGCCAACGCCATCTACTTCAAGGGCAAGTGGGCGAAGCCATTCGCCGGGTATCTCACCCAGGACGACAAGTTCCACCGCCTCGACGGCACCGCCGTCGATGCCCCCTTCATGTGCGGTCAAGGCAGGCACAACATCGCTTGCCACCATGGCTTCAAGGTGCTCCAGCTCCGCTACGAGGAAGGGCACGGCCCCTTGCTGCCCCAGCAGCCGGCTCTAGCTCCGACGGCGGCGCCGATCTACTCGATGTGCGTCTTCCTCCCGGATGCGCGCCGCGGGCTTTGGCGGCTCACCGACAAGATCGCGTGCAACCCCGACTTTCTGCGCAAGCACCTGCCGAGGGACACTGTCCTGGTCGGCGACTTCCGGTTGCCTAAATTCAAAGTCACCTTCGACATGACGATGAACGACGTTCTCCAGGAGATGGGGGTCAAGGAGGCGTTCGAGCTGGGGAAGGCCGACCTGTCCGACATGGCGGAGGACGGCGCAAGGAGGAAGCTGGCACTAGAGCAGGTGATCCACAGGGCCGTCATCGAGGTGAACGAGGAAGGCACCGAGGCGGCGGCCGCCACTTGCATGACGCGCCTTGGGTGCACACCGCACTCGCTGCCGCTTGCACCGTGTGTGGACTTCGTGGCTGACCATCCATTTGCCTTCTTCATCGTCGAGGAGGTGTCAGGCGCGATTCTGTTTGCGGGGCACGTCCTTGATCCCACCATCAAGTGA

>TraesCS2B02G050210-protien

MESVKEFALRRFPKLGQWFYTEADADQAAPGDGLQAFSLGLNKRLAHDAGRRSNLVFSPLSVYAGLSLVAAGARDRTLDELLAVLGAPSRDFLAGHVRALAEQALTDQSKTGGPRVSFACGVWHDRTMPIRPAYRDAAESFKAVARAVNFRQKPEEAREEINAWVSASTDGLIPSILSRGALSDLTDLVLANAIYFKGKWAKPFAGYLTQDDKFHRLDGTAVDAPFMCGQGRHNIACHHGFKVLQLRYEEGHGPLLPQQPALAPTAAPIYSMCVFLPDARRGLWRLTDKIACNPDFLRKHLPRDTVLVGDFRLPKFKVTFDMTMNDVLQEMGVKEAFELGKADLSDMAEDGARRKLALEQVIHRAVIEVNEEGTEAAAATCMTRLGCTPHSLPLAPCVDFVADHPFAFFIVEEVSGAILFAGHVLDPTIK*

Using the TraesCS2A02G036700 sequence, we searched the wheat TGACv1 scaffold and found the homologous gene on chromosome 2D, the protein product of which was named WSZ13-D. This sequence was then confirmed in IWGSC RefSeq v1.0. The blast result is below.

>WSZ13-D-CDS on chr2D [
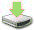
](https://urgi.versailles.inrae.fr/download/iwgsc/IWGSC_RefSeq_Assemblies/v1.0/) [
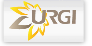
](http://wheat-urgi.versailles.inrae.fr/Seq-Repository/Assemblies)

Length=651852609

Score = 1508 bits (1672), Expect = 0.0

Identities = 836/836 (100%), Gaps = 0/836 (0%)

Strand=Plus/Plus

Query 464 AGCCGGAGGAGGCTACGGAGGAGATCAACGCATGGGTGTCGGCGTCGACGGACGGCCTCA 523

||||||||||||||||||||||||||||||||||||||||||||||||||||||||||||

Sbjct 13632968 AGCCGGAGGAGGCTACGGAGGAGATCAACGCATGGGTGTCGGCGTCGACGGACGGCCTCA 13633027

Query 524 TCCCCTCCATCCTTAGCCGGGGCGCGCTGTCAGACCTCACCGACCTGGTGCTCGCCAACG 583

||||||||||||||||||||||||||||||||||||||||||||||||||||||||||||

Sbjct 13633028 TCCCCTCCATCCTTAGCCGGGGCGCGCTGTCAGACCTCACCGACCTGGTGCTCGCCAACG 13633087

Query 584 CCATCTACTTTAAGGGCAAGTGGGCGAAGCCATTCGCCGGGTATCTCACCCAGCACGACA 643

||||||||||||||||||||||||||||||||||||||||||||||||||||||||||||

Sbjct 13633088 CCATCTACTTTAAGGGCAAGTGGGCGAAGCCATTCGCCGGGTATCTCACCCAGCACGACA 13633147

Query 644 AGTTCCACCGCCTTGACGGCACCGCCGTCGATGCCCCCTTCATGCGCGGTCTCGGCAGCC 703

||||||||||||||||||||||||||||||||||||||||||||||||||||||||||||

Sbjct 13633148 AGTTCCACCGCCTTGACGGCACCGCCGTCGATGCCCCCTTCATGCGCGGTCTCGGCAGCC 13633207

Query 704 ACAACATCGCCTGCCACGATGGCTTCAAGGTGCTGCAGCTCCGCTACGAGGAAGGGCACG 763

||||||||||||||||||||||||||||||||||||||||||||||||||||||||||||

Sbjct 13633208 ACAACATCGCCTGCCACGATGGCTTCAAGGTGCTGCAGCTCCGCTACGAGGAAGGGCACG 13633267

Query 764 GCCCCTTGCTGCCCCAGCCACCAGCTCTAGCTCCCACGCCTGTGCCGATCTACTCGATGT 823

||||||||||||||||||||||||||||||||||||||||||||||||||||||||||||

Sbjct 13633268 GCCCCTTGCTGCCCCAGCCACCAGCTCTAGCTCCCACGCCTGTGCCGATCTACTCGATGT 13633327

Query 824 GCGTCTTCCTCCCCGATGCGCGCCGCGGGCTGTGGCGGCTCACCGACAAGATCGCGTGCA 883

||||||||||||||||||||||||||||||||||||||||||||||||||||||||||||

Sbjct 13633328 GCGTCTTCCTCCCCGATGCGCGCCGCGGGCTGTGGCGGCTCACCGACAAGATCGCGTGCA 13633387

Query 884 ACCCCGACTTTCTGCGCAAGCACTTGCCGAGGAACAGTGTCCTGGTCGGCGACTTCCGGC 943

||||||||||||||||||||||||||||||||||||||||||||||||||||||||||||

Sbjct 13633388 ACCCCGACTTTCTGCGCAAGCACTTGCCGAGGAACAGTGTCCTGGTCGGCGACTTCCGGC 13633447

Query 944 TACCTAAATTCAAGGTAACCTTCGGCATGACGATGAACGACGTACTCCAGGAAATGGGGG 1003

||||||||||||||||||||||||||||||||||||||||||||||||||||||||||||

Sbjct 13633448 TACCTAAATTCAAGGTAACCTTCGGCATGACGATGAACGACGTACTCCAGGAAATGGGGG 13633507

Query 1004 TCAAGGAGGCGTTCGAGCTGGGGAAGGCCGACCTGTCCAACATGGTGGAGAACGGCGGAA 1063

||||||||||||||||||||||||||||||||||||||||||||||||||||||||||||

Sbjct 13633508 TCAAGGAGGCGTTCGAGCTGGGGAAGGCCGACCTGTCCAACATGGTGGAGAACGGCGGAA 13633567

Query 1064 GGAGGAAGATGGCGCTGGAAAAGGTGATCCACATGGCCGTCATCGAGGTGAACGAGGAAG 1123

||||||||||||||||||||||||||||||||||||||||||||||||||||||||||||

Sbjct 13633568 GGAGGAAGATGGCGCTGGAAAAGGTGATCCACATGGCCGTCATCGAGGTGAACGAGGAAG 13633627

Query 1124 GCACAGAGGCGGCGGCCGCCACTTACATGACGCGCCTTGGATGCACACCGGACTCGCGGC 1183

||||||||||||||||||||||||||||||||||||||||||||||||||||||||||||

Sbjct 13633628 GCACAGAGGCGGCGGCCGCCACTTACATGACGCGCCTTGGATGCACACCGGACTCGCGGC 13633687

Query 1184 CGCCTGCACCGTGTGTGGACTTCGTGGCTGACCATCCATTTGCCTTCTTCATCGTCGAGG 1243

||||||||||||||||||||||||||||||||||||||||||||||||||||||||||||

Sbjct 13633688 CGCCTGCACCGTGTGTGGACTTCGTGGCTGACCATCCATTTGCCTTCTTCATCGTCGAGG 13633747

Query 1244 AGGTGTCAGGCGCGATTCTGTTCGCGGGGCATGTCCTTGATCCCACCATCAAGTGA 1299

||||||||||||||||||||||||||||||||||||||||||||||||||||||||

Sbjct 13633748 AGGTGTCAGGCGCGATTCTGTTCGCGGGGCATGTCCTTGATCCCACCATCAAGTGA 13633803

Score = 839 bits (930), Expect = 0.0

Identities = 465/465 (100%), Gaps = 0/465 (0%)

Strand=Plus/Plus

Query 1 ATGGAATCTATGAAGGAGTTCGCCCTCCGCCGTTTTCCGAAGCTAGGCAGATGGTTCTGC 60

||||||||||||||||||||||||||||||||||||||||||||||||||||||||||||

Sbjct 13632381 ATGGAATCTATGAAGGAGTTCGCCCTCCGCCGTTTTCCGAAGCTAGGCAGATGGTTCTGC 13632440

Query 61 ACCGAGGCCGACGCCGGCGCCGATCAGGCCGTTCCCAGCGATGGCCTGCAGGCCTTCTCC 120

||||||||||||||||||||||||||||||||||||||||||||||||||||||||||||

Sbjct 13632441 ACCGAGGCCGACGCCGGCGCCGATCAGGCCGTTCCCAGCGATGGCCTGCAGGCCTTCTCC 13632500

Query 121 CTCGGCCTGAATAAGCGCCTCGCGCACGACGCCGGCAGGAGGAGCAACCTGGTCTTCTCG 180

||||||||||||||||||||||||||||||||||||||||||||||||||||||||||||

Sbjct 13632501 CTCGGCCTGAATAAGCGCCTCGCGCACGACGCCGGCAGGAGGAGCAACCTGGTCTTCTCG 13632560

Query 181 CCGCTGTCCGTCTACGCCGGGCTCTCGCTGGTAGCCGCGGGCGCCCGCGACCGCACCCTC 240

||||||||||||||||||||||||||||||||||||||||||||||||||||||||||||

Sbjct 13632561 CCGCTGTCCGTCTACGCCGGGCTCTCGCTGGTAGCCGCGGGCGCCCGCGACCGCACCCTC 13632620

Query 241 GACGAGCTGCTCGTTGTCCTCGGCGCGCCGTCGCGGGACTTCCTCGCCGGCGACGTCCGT 300

||||||||||||||||||||||||||||||||||||||||||||||||||||||||||||

Sbjct 13632621 GACGAGCTGCTCGTTGTCCTCGGCGCGCCGTCGCGGGACTTCCTCGCCGGCGACGTCCGT 13632680

Query 301 GCGCTGGCCGAGCAGGCCCTCACGGACCAGTCCAAGACCGGCGGCCCGCGCATCAGCTTC 360

||||||||||||||||||||||||||||||||||||||||||||||||||||||||||||

Sbjct 13632681 GCGCTGGCCGAGCAGGCCCTCACGGACCAGTCCAAGACCGGCGGCCCGCGCATCAGCTTC 13632740

Query 361 GCGTGCGGCGTGTGGCATGACCGGACCATGCCCATCCGCCCCGCCTACCGCGATGCCGCC 420

||||||||||||||||||||||||||||||||||||||||||||||||||||||||||||

Sbjct 13632741 GCGTGCGGCGTGTGGCATGACCGGACCATGCCCATCCGCCCCGCCTACCGCGATGCCGCC 13632800

Query 421 GAATCCTTCAAGGCCGTCGCCCGCGCCGTCAACTTCCGCCAAAAG 465

|||||||||||||||||||||||||||||||||||||||||||||

Sbjct 13632801 GAATCCTTCAAGGCCGTCGCCCGCGCCGTCAACTTCCGCCAAAAG 13632845

In refseq v1.0, the gene encoding WSZ13-D is on 2D: 13632381.. 13632845, 13632970.. 13633803. A gene was annotated in this region, TraesCS2D02G036000:

>chr2D chr2D:13633576..13633803 (+ strand) class=mRNA length=228

ATGGCGCTGGAAAAGGTGATCCACATGGCCGTCATCGAGGTGAACGAGGAAGGCACAGAGGCGGCGGCCGCCACTTACATGACGCGCCTTGGATGCACACCGGACTCGCGGCCGCCTGCACCGTGTGTGGACTTCGTGGCTGACCATCCATTTGCCTTCTTCATCGTCGAGGAGGTGTCAGGCGCGATTCTGTTCGCGGGGCATGTCCTTGATCCCACCATCAAGTGA

We used the name “TraesCS2D02G036000*” as we corrected this annotation.

>TraesCS2D02G036000*-CDS

ATGGAATCTATGAAGGAGTTCGCCCTCCGCCGTTTTCCGAAGCTAGGCAGATGGTTCTGCACCGAGGCCGACGCCGGCGCCGATCAGGCCGTTCCCAGCGATGGCCTGCAGGCCTTCTCCCTCGGCCTGAATAAGCGCCTCGCGCACGACGCCGGCAGGAGGAGCAACCTGGTCTTCTCGCCGCTGTCCGTCTACGCCGGGCTCTCGCTGGTAGCCGCGGGCGCCCGCGACCGCACCCTCGACGAGCTGCTCGTTGTCCTCGGCGCGCCGTCGCGGGACTTCCTCGCCGGCGACGTCCGTGCGCTGGCCGAGCAGGCCCTCACGGACCAGTCCAAGACCGGCGGCCCGCGCATCAGCTTCGCGTGCGGCGTGTGGCATGACCGGACCATGCCCATCCGCCCCGCCTACCGCGATGCCGCCGAATCCTTCAAGGCCGTCGCCCGCGCCGTCAACTTCCGCCAAAAGCCGGAGGAGGCTACGGAGGAGATCAACGCATGGGTGTCGGCGTCGACGGACGGCCTCATCCCCTCCATCCTTAGCCGGGGCGCGCTGTCAGACCTCACCGACCTGGTGCTCGCCAACGCCATCTACTTTAAGGGCAAGTGGGCGAAGCCATTCGCCGGGTATCTCACCCAGCACGACAAGTTCCACCGCCTTGACGGCACCGCCGTCGATGCCCCCTTCATGCGCGGTCTCGGCAGCCACAACATCGCCTGCCACGATGGCTTCAAGGTGCTGCAGCTCCGCTACGAGGAAGGGCACGGCCCCTTGCTGCCCCAGCCACCAGCTCTAGCTCCCACGCCTGTGCCGATCTACTCGATGTGCGTCTTCCTCCCCGATGCGCGCCGCGGGCTGTGGCGGCTCACCGACAAGATCGCGTGCAACCCCGACTTTCTGCGCAAGCACTTGCCGAGGAACAGTGTCCTGGTCGGCGACTTCCGGCTACCTAAATTCAAGGTAACCTTCGGCATGACGATGAACGACGTACTCCAGGAAATGGGGGTCAAGGAGGCGTTCGAGCTGGGGAAGGCCGACCTGTCCAACATGGTGGAGAACGGCGGAAGGAGGAAGATGGCGCTGGAAAAGGTGATCCACATGGCCGTCATCGAGGTGAACGAGGAAGGCACAGAGGCGGCGGCCGCCACTTACATGACGCGCCTTGGATGCACACCGGACTCGCGGCCGCCTGCACCGTGTGTGGACTTCGTGGCTGACCATCCATTTGCCTTCTTCATCGTCGAGGAGGTGTCAGGCGCGATTCTGTTCGCGGGGCATGTCCTTGATCCCACCATCAAGTGA

>TraesCS2D02G036000*-protein

MESMKEFALRRFPKLGRWFCTEADAGADQAVPSDGLQAFSLGLNKRLAHDAGRRSNLVFSPLSVYAGLSLVAAGARDRTLDELLVVLGAPSRDFLAGDVRALAEQALTDQSKTGGPRISFACGVWHDRTMPIRPAYRDAAESFKAVARAVNFRQKPEEATEEINAWVSASTDGLIPSILSRGALSDLTDLVLANAIYFKGKWAKPFAGYLTQHDKFHRLDGTAVDAPFMRGLGSHNIACHDGFKVLQLRYEEGHGPLLPQPPALAPTPVPIYSMCVFLPDARRGLWRLTDKIACNPDFLRKHLPRNSVLVGDFRLPKFKVTFGMTMNDVLQEMGVKEAFELGKADLSNMVENGGRRKMALEKVIHMAVIEVNEEGTEAAAATYMTRLGCTPDSRPPAPCVDFVADHPFAFFIVEEVSGAILFAGHVLDPTIK
